# Aptamer‐Directed Porous DNA Nanocomposite Hydrogel for Active Pulp Preservation: Immunomodulation, Stem Cell Recruitment and Reparative Dentinogenesis

**DOI:** 10.1002/advs.75497

**Published:** 2026-05-07

**Authors:** Luhui Cai, Huan Yu, Zihan Ni, Kan Chen, I‐Chen Tsai, Zeyu Bao, Shuo Sun, Weichang Li, Yunhua Chen, Qiong Xu

**Affiliations:** ^1^ Hospital of Stomatology Sun Yat‐sen University Guangzhou China; ^2^ Guangdong Provincial Key Laboratory of Stomatology Guangzhou China; ^3^ Guanghua School of Stomatology Sun Yat‐sen University Guangzhou China; ^4^ School of Materials Science and Engineering South China University of Technology Guangzhou China; ^5^ National Engineering Research Center for Tissue Restoration and Reconstruction South China University of Technology Guangzhou China; ^6^ Guangdong Provincial Key Laboratory of Biomedical Engineering South China University of Technology Guangzhou China

**Keywords:** air‐in‐water emulsions, aptamer, DNA hydrogels, pulpitis, reparative dentin

## Abstract

Vital pulp therapy (VPT) is a conservative alternative to root canal treatment that preserves the vitality of the pulp–dentin complex, yet its clinical outcomes remain inconsistent due to persistent inflammation and insufficient endogenous stem cell participation. Here, we report an injectable, aptamer‐functionalized porous double‐network hydrogel (DGDL‐Apt) for active pulp preservation through coordinated immune–redox microenvironment reprogramming and targeted stem cell recruitment. Fabricated via an air‐in‐water emulsion strategy, the hydrogel integrates a dopamine‐modified gelatin methacryloyl network with a supramolecular DNA–Laponite assembly, forming a mechanically robust and interconnected porous structure. Dopamine‐derived catechol motifs restore redox homeostasis and attenuate inflammation, promoting macrophage polarization toward a reparative M2 phenotype. Meanwhile, sustained release of Laponite‐derived ions enhances odontogenic differentiation, and CD29‐targeting aptamers enable precise in situ recruitment of endogenous dental pulp stem cells. The DGDL‐Apt hydrogel suppresses oxidative stress, reprograms macrophage phenotype, and promotes odontogenic differentiation in vitro. In a pulpitis rat model, it achieves rapid inflammation resolution, efficient stem cell enrichment, and formation of a continuous reparative dentin bridge. This work advances VPT from passive pulp‐capping toward an active regenerative strategy and provides a general design principle for microenvironment‐driven tissue preservation.

## Graphical abstract

This study presents an injectable DNA‐based porous hydrogel integrating catechol motifs and targeting aptamers for pulpitis management. Upon in situ crosslinking, the scaffold actively recruits endogenous dental pulp stem cells, restores redox homeostasis, and modulates immune responses. This multi‐pronged strategy successfully resolves inflammation and induces robust reparative dentinogenesis, offering a promising solution for vital pulp preservation.

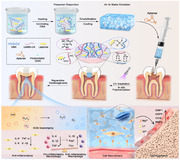

## 1 Introduction

Pulpitis, predominantly induced by bacterial infiltration resulting from deep caries, trauma, or mechanical irritation, remains one of the most prevalent inflammatory diseases in endodontics [1]. Confined within the rigid dentin walls, the dental pulp is a highly specialized, vascularized and innervated tissue responsible for nutrition, sensory transmission and innate immune defense [2]. Preservation of pulp vitality is therefore essential for maintaining tooth integrity and long‐term function. However, root canal treatment (RCT) continues to be the standard intervention for irreversible pulpitis, despite requiring complete removal of the pulp tissue, which permanently abolishes neurovascular supply, compromises biomechanical integrity and markedly increases the risk of long‐term tooth fracture [3]. Emerging histological and microbiological evidence has demonstrated that, during the early stage of reversible pulpitis, inflammation and bacterial colonization are frequently restricted to the coronal pulp, while the radicular pulp remains vital and sterile [4, 5]. These findings, together with the principles of minimally invasive dentistry, have driven a approach shift toward vital pulp therapy (VPT), which aims to preserve the residual pulp tissue and its regenerative potential rather than replacing it with inert filling materials [6]. However, despite its conceptual appeal, the long‐term success of VPT remains inconsistent, indicating that preservation of pulp vitality requires more than simple physical sealing of the exposure site.

Beyond the operator's technical proficiency, the clinical outcome of VPT is fundamentally dictated by the biological performance of the pulp‐capping material, which should ideally function not only as a physical barrier but also as an active regulator of the local inflammatory microenvironment [6, 7]. Calcium silicate–based bioceramics, including mineral trioxide aggregate (MTA) and iRoot BP Plus, are currently regarded as the clinical gold standard for pulp capping [8]. However, their therapeutic efficacy remains limited [9]. Although their high alkalinity provides a degree of antibacterial activity, these materials fail to address excessive reactive oxygen species (ROS) accumulation, a hallmark of inflamed pulp tissue that disrupts redox homeostasis and perpetuates inflammatory damage [9, 10, 11]. Notably, the initial contact between alkaline bioceramics and pulp tissue may even provoke transient inflammatory responses, potentially compromising pulp preservation [9]. Moreover, successful dentin–pulp regeneration critically depends on the presence and functional competence of dental pulp stem cells (DPSCs) [9]. In inflamed pulp, however, the endogenous pool of DPSCs is often insufficient or functionally impaired, and currently available bioceramics lack specific bioactive cues capable of actively recruiting or retaining endogenous DPSCs at the injury site. As a result, existing VPT materials largely operate in a passive manner, sealing the pulp exposure area while failing to actively reprogram the hostile inflammatory microenvironment into one that supports tissue regeneration.

Injectable hydrogels have emerged as promising candidates for VPT, primarily because their minimally invasive delivery enables immediate intervention within the confined pulp chamber while conforming precisely to irregular cavity geometries [12, 13]. Beyond clinical handling advantages, such injectability is particularly valuable for early‐stage pulpitis, where timely modulation of the local microenvironment is critical for preventing irreversible inflammatory progression. Among available hydrogel matrices, gelatin methacryloyl (GelMA) is widely employed owing to its excellent biocompatibility, tunable crosslinking density and ease of clinical application [14]. In recent years, DNA supramolecular hydrogels have garnered widespread attention due to their unique programmability, reversible assembly and capacity for precise molecular recognition mediated by complementary base pairing [15, 16, 17, 18, 19]. Incorporation of DNA motifs into GelMA networks offers a powerful strategy to introduce bio‐instructive functionality, enabling dynamic and targeted biological interactions that extend beyond the passive structural role of conventional hydrogels [20, 21, 22, 23, 24]. Such hybrid systems therefore provide an attractive platform for actively regulating cell behavior within inflamed tissues.

However, most conventional injectable hydrogels form dense and poorly interconnected networks, which function primarily as physical barriers rather than regenerative microenvironments [25, 26]. This compact architecture severely limits oxygen and nutrient diffusion, impairs metabolic waste clearance, and restricts immune cell turnover and stem cell infiltration—factors that are particularly detrimental in inflamed pulp tissue where redox imbalance and immune dysregulation already prevail [27]. To overcome these limitations, emulsion templating has emerged as an effective approach for constructing highly porous, interconnected hydrogel scaffolds [28, 29]. Specifically, the air‐in‐water templating strategy exploits the amphiphilic nature of pre‐polymers to stabilize microbubbles under high‐shear conditions, which subsequently act as sacrificial templates to generate interconnected macroporous networks upon crosslinking [29, 30, 31, 32, 33]. Importantly, such porous architectures more closely recapitulate the dynamic features of native extracellular matrix (ECM), facilitating rapid molecular exchange, immune cell trafficking and cellular infiltration [21, 34, 35, 36, 37]. These characteristics suggest that macroporous injectable hydrogels may serve not merely as space‐filling materials, but as active platforms capable of supporting microenvironmental reprogramming during vital pulp preservation.

Nevertheless, an optimized physical scaffold alone is insufficient for pulp‐dentin regeneration, the participation of functional dental pulp stem cells is also essential [38, 39]. While exogenous stem cell transplantation has been widely explored, it still faces significant disadvantages, including immune rejection, ethical concerns and high costs [40]. Alternatively, the in situ recruitment of the host's own stem cells offers a more clinically promising approach [32]. Aptamers, defined as short single‐stranded oligonucleotides, have emerged as ideal targeting ligands due to their exceptional specificity and affinity [41]. Acting as “cellular magnets”, they enable the precise capture and enrichment of endogenous targeted cells [42, 43]. Accordingly, a regenerative strategy that actively mobilizes endogenous DPSCs while simultaneously reprogramming the hostile inflammatory microenvironment is highly desirable for effective vital pulp therapy.

To construct a bio‐instructive matrix, Laponite was also incorporated into the hydrogel. The anisotropic charge distribution of Laponite enables its uniform dispersion and significantly enhances the precursor's rheological profile [44]. Even at low concentrations, it imparts superior thixotropy, which is critical for preserving the emulsion‐templated porous architecture during the sol‐to‐gel transition [45]. The dynamic non‐covalent interactions between the nanodisks and the polymer chains establish a sacrificial bonding network, yielding a high‐strength nanocomposite capable of withstanding the mechanical stresses. In addition, Laponite serves as a potent, cell‐free odontogenic inducer [46]. The sustained release of its constitutive bioactive ions Li^+^, Mg^2+^ and Si^4+^ can directly upregulate odontogenic markers like DSPP and DMP‐1 and trigger odontogenic differentiation of DPSCs [46].

Herein, we report a multifunctional injectable double‐network hydrogel (DGDL‐Apt) designed to enable active pulp preservation through coordinated microenvironmental reprogramming and targeted endogenous stem cell recruitment. The scaffold is fabricated via an air‐in‐water emulsion templating strategy, generating an interconnected macroporous architecture that integrates a covalently crosslinked gelatin methacryloyl–dopamine (GelMA–DA) network with a supramolecular DNA–Laponite assembly. This double‐network design provides sufficient mechanical robustness to withstand pulpal pressure while maintaining a permissive porous microenvironment for molecular exchange and cellular infiltration. Functionally, dopamine moieties act as redox regulators to scavenge ROS and attenuate inflammatory stress, while matrix‐anchored aptamers serve as biological homing cues to selectively recruit endogenous DPSCs. Meanwhile, bioactive ions released from Laponite further promote odontogenic differentiation, collectively facilitating the formation of a reparative dentin bridge. The physicochemical properties, immunoregulatory effects and regenerative performance of DGDL‐Apt were systematically evaluated in vitro and in a rat model of lipopolysaccharide‐induced pulpitis. Together, this work establishes an active, microenvironment‐oriented pulp capping strategy that advances vital pulp therapy beyond passive sealing toward functional tissue preservation, offering a promising new approach for the management of pulpitis.

## 2 Results and Discussion

### 2.1 Fabrication of DGDL Hydrogel

**FIGURE 1:**
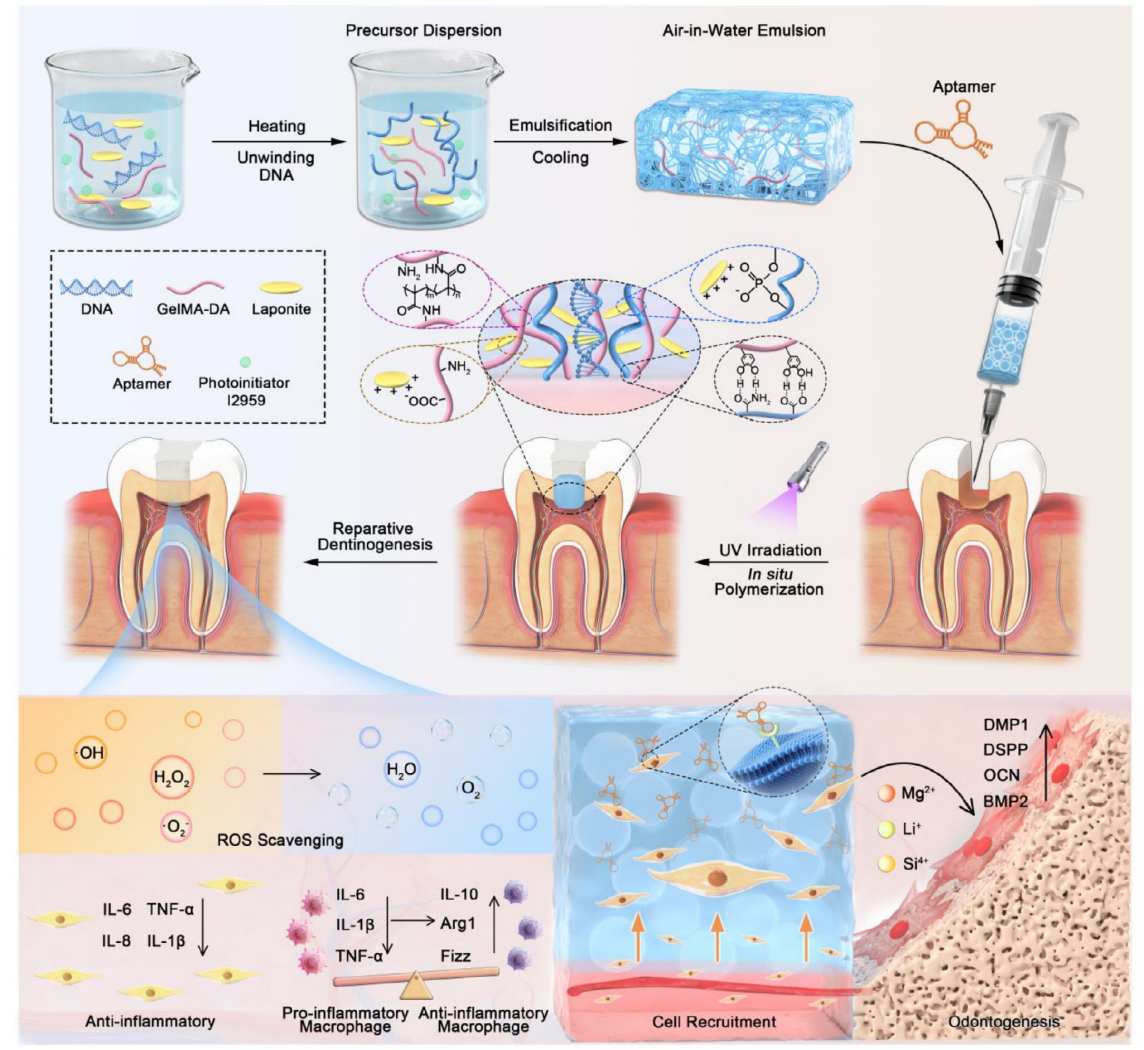
Schematic illustration of the design and therapeutic mechanism of the DGDL‐Apt hydrogel. The DGDL precursor is prepared via facile shear‐emulsification, integrating DNA, catechol‐modified GelMA (GelMA‐DA) and Laponite clay nanosheets to form an air‐in‐water emulsion. After conjugation with CD29‐targeting aptamers, the injectable precursor is delivered into the exposed pulp cavity and photo‐crosslinked in situ under UV irradiation. The DGDL‐Apt hydrogel functions as a multifunctional platform: (i) ROS scavenging and active redox homeostasis restoration via catechol antioxidants; (ii) Immunomodulation through reprogramming of macrophage polarization from a pro‐inflammatory M1 to a pro‐regenerative M2 phenotype; and (iii) Aptamer‐mediated active recruitment of endogenous dental pulp stem cells (DPSCs) to the injury site and subsequent promotion of their odontogenic differentiation.

The design of the DGDL‐Apt hydrogel was guided by the central premise that successful vital pulp therapy requires coordinated modulation of the inflammatory microenvironment and restoration of endogenous regenerative capacity, rather than simple physical sealing. To this end, a multifunctional double‐network architecture was constructed by integrating complementary chemical, supramolecular and inorganic components within a single injectable scaffold (Figure 1).

The primary covalent network was formed using gelatin methacryloyl functionalized with dopamine (GelMA‐DA), which provides both structural integrity and redox‐regulatory functionality. Successful conjugation of dopamine onto the GelMA backbone was confirmed by ^1^H NMR spectroscopy (Figure S1), where the emergence of a characteristic resonance at δ = 2.87 ppm corresponded to the methylene protons of dopamine. FTIR analysis further corroborated this modification (Figure S2), revealing a distinct absorption band at 870 cm^−^^1^ attributable to out‐of‐plane C–H bending of the aromatic catechol ring, while preserving the characteristic amide and hydroxyl signals of GelMA. Quantitative analysis indicated degrees of substitution of 80.1% for methacrylate groups and 20.5% for catechol moieties [47], enabling robust photocrosslinking while retaining sufficient catechol content for effective redox modulation. To introduce dynamic adaptability and biological programmability, DNA strands were embedded within the GelMA‐DA matrix to form a secondary supramolecular network. Owing to their precise and reversible complementary base pairing, DNA strands establish extensive hydrogen‐bonded physical crosslinks [17, 18, 19], endowing the hydrogel with dynamic reversibility and the capacity to respond to the evolving pulp microenvironment. This supramolecular network not only contributes to mechanical resilience but also provides molecular anchoring sites for subsequent aptamer functionalization, thereby linking structural design directly to biological function.

Laponite nanoplates were further incorporated to simultaneously enhance mechanical robustness and odontogenic bioactivity. Benefiting from their anisotropic disc‐like morphology and dual‐charge surface distribution, Laponite acts as a multifunctional physical crosslinker within the composite network [44, 48]. Transmission electron microscopy (TEM) revealed that pristine Laponite exhibited discrete nanodisc structures with well‐defined edges (Figure S3A), whereas within the DGDL precursor, the nanoplates were homogeneously dispersed throughout the DNA–GelMA‐DA matrix without noticeable aggregation (Figure S3B). This uniform distribution was further confirmed by EDS elemental mapping (Figure S4), where Mg and Si signals characteristic of Laponite were spatially co‐localized with C, O and Na elements of the polymeric matrix. Such a well‐interconnected organic–inorganic hybrid network is critical for effective stress transfer and mechanical stability. According to stress‐transfer theory, uniformly distributed rigid nanoplatelets can dissipate applied mechanical energy and mitigate localized stress concentrations, thereby reinforcing the hydrogel against deformation under pulpal pressure [49]. Consistent with this mechanism, zeta potential analysis revealed a pronounced shift in surface charge upon network integration (Figure S5). While pure DNA exhibited a strongly negative potential (−53.2 mV), and Laponite and GelMA displayed moderate negative charges (−22.1 mV and −10.4 mV, respectively), the DGDL hydrogel exhibited a zeta potential of −55.8 mV. This value closely matched that of DNA, confirming successful incorporation of DNA strands and the formation of a stable, electrostatically reinforced supramolecular network. Collectively, this double‐network design establishes a mechanically robust yet dynamically adaptable scaffold that provides the structural and biochemical foundation for subsequent redox regulation and immune reprogramming and targeted endogenous stem cell recruitment, thereby enabling active pulp preservation.

### 2.2 Injectability and Structural Analysis of DGDL Hydrogel

**FIGURE 2:**
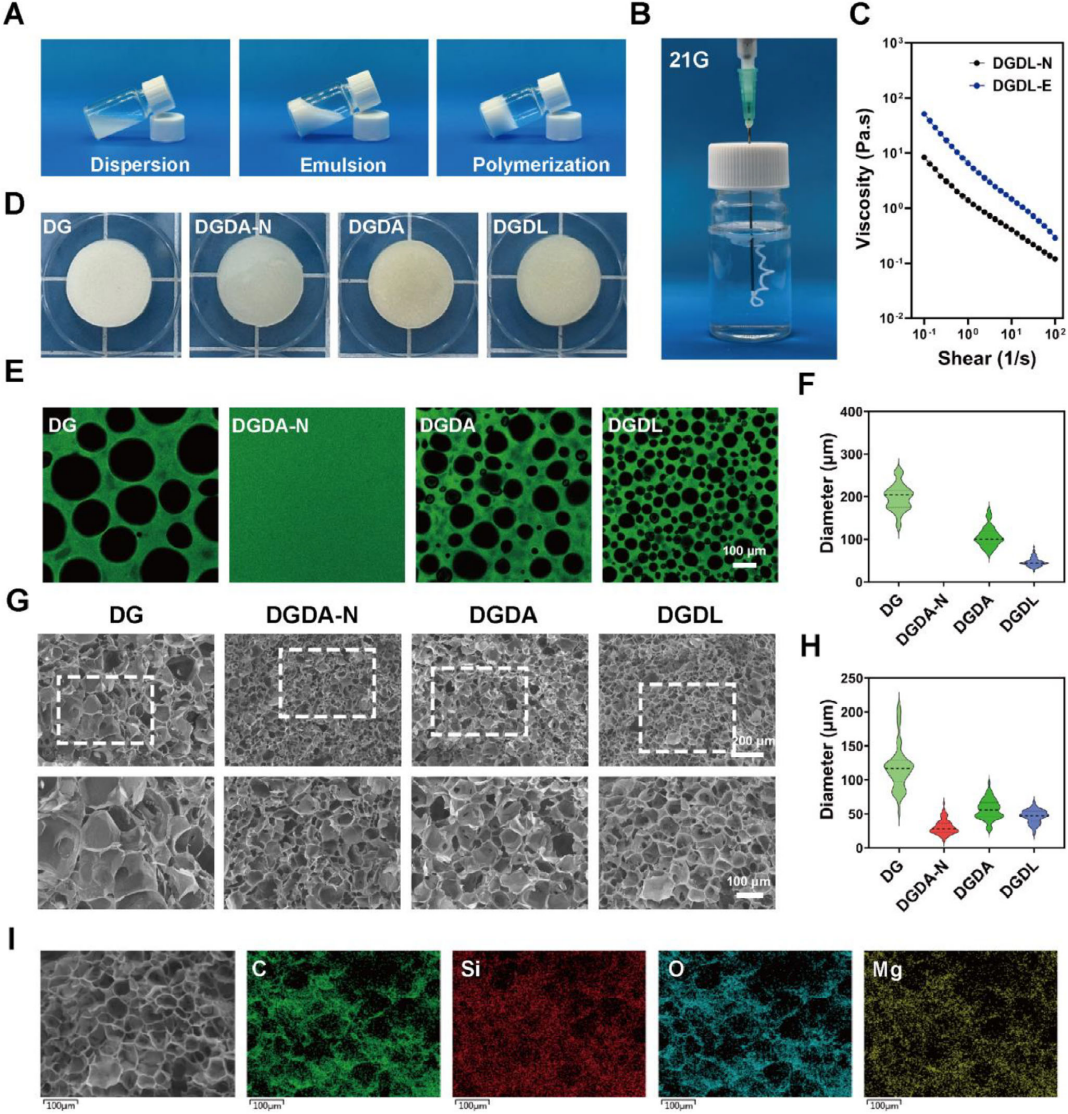
Fabrication and microstructural analysis of the DGDL hydrogel. (A) Macroscopic observation of the dispersion precursor upon emulsion formation and subsequent polymerization. (B) Injectability test through a 21G needle and (C) shear‐thinning viscosity profiles of the DGDL precursor. (D) Optical images exhibiting the gross morphology of different hydrogel groups (DG, DGDA‐N, DGDA and DGDL). (E) 3D reconstruction of the porous network via CLSM (stained with FITC) and (F) the corresponding pore size distribution quantified by violin plots. (G) Representative SEM micrographs of the hydrogel microarchitecture and (H) Quantitative analysis of pore diameters. (I) SEM‐EDS elemental mapping (C, Si, O, Mg) confirming the homogeneous dispersion of components within the DGDL hydrogel.

Unlike conventional dense hydrogels that primarily function as physical barriers, the DGDL hydrogel was engineered using an air‐in‐water (A/W) emulsion templating strategy to establish a hierarchically interconnected porous architecture capable of supporting active microenvironmental regulation [29, 34]. As illustrated in Figure 2A, the initially homogeneous DGDL dispersion underwent rapid emulsification under high‐speed shear, forming a stable milky‐white A/W emulsion accompanied by a noticeable volumetric expansion. The emulsion was high stable without de‐emulsification observed for several days, might could attribute to the good emulsifying capability of GelMA stabilizer and the high viscosity of the aqueous continuous phase [29]. Subsequent UV‐triggered photopolymerization yielded a mechanically robust hydrogel with well‐preserved structural integrity, indicating successful fixation of the emulsion‐derived porous network [29, 34]. Such macroporous architecture is particularly relevant in the context of inflamed pulp tissue, where restricted oxygen diffusion, impaired metabolite clearance, and limited immune cell trafficking contribute to persistent inflammation. By introducing interconnected pores, the DGDL hydrogel provides continuous diffusion pathways that facilitate molecular exchange, cellular infiltration and waste removal, thereby creating a permissive physical framework for microenvironmental reprogramming. The shape adaptability of the DGDL hydrogel was further evaluated to assess its capacity to conform to irregular pulp exposure sites. As shown in Figure S6A (Supporting Information), the photocurable emulsion precursor could be molded into complex geometries, including tooth‐like structures, with high shape fidelity. This conformability ensures intimate contact with the dentin walls, minimizing marginal gaps that could otherwise permit bacterial infiltration and perpetuate the inflammatory response.

Injectability is a prerequisite for early‐stage intervention in vital pulp therapy. As demonstrated In Figure 2B and Figure S6B, the DGDL emulsion precursor could be continuously extruded through standard 21G and 24G needles into aqueous environments, forming coherent filaments that retained structural integrity prior to curing. This excellent injectability was further corroborated in Figure S6C (Supporting Information), where the hydrogel was smoothly extruded to write the letters “SYSU”. Rheological analysis revealed that although the emulsified precursor (DGDL‐E) exhibited higher viscosity due to the structured foam architecture, it retained pronounced shear‐thinning behavior similar to the non‐porous control (DGDL‐N) (Figure 2C). This shear‐thinning characteristic enables facile injection under applied stress while allowing rapid structural recovery after extrusion, ensuring efficient filling of confined and irregular pulp defects before in situ solidification.

To delineate the respective contributions of emulsification, dopamine functionalization and Laponite incorporation, four formulations were systematically compared: DG (emulsified DNA‐GelMA), DGDA‐N (non‐porous DNA‐GelMA‐DA), DGDA (emulsified DNA‐GelMA‐DA) and DGDL (emulsified nanocomposite). Macroscopically, all emulsified hydrogels exhibited a characteristic opaque appearance due to light scattering from uniformly distributed air microbubbles, whereas the non‐porous DGDA‐N hydrogel remained optically transparent (Figure 2D). Confocal laser scanning microscopy (CLSM) revealed that, under hydrated conditions, the DGDA‐N group formed a continuous, dense matrix, whereas all emulsified groups displayed well‐defined circular pore structures with high interconnectivity (Figure 2E). Quantitative pore size analysis (Figure 2F) indicated that dopamine functionalization reduced pore dimensions and improved structural regularity, while the DGDL hydrogel exhibited the most uniform pore size distribution among all emulsified groups. The significant reduction and enhanced uniformity in pore size observed in the DGDL hydrogel can be attributed to a synergistic effect by the Laponite nanodisks. The electrostatic interactions between the nanodisks and DNA molecules elevate the viscosity and yield stress of the continuous aqueous phase. During emulsification, this viscoelastic continuous phase can transmit mechanical shear stress to the dispersed phase, promoting the fragmentation of air into much smaller micro‐droplets [50]. Besides, the Laponite nanodisks might act as effective solid co‐stabilizers [51, 52]. They spontaneously adsorb at the newly formed air‐water interfaces, effectively inhibits subsequent droplet coalescence. Together, these effects preserve pore interconnectivity and prevent structural collapse, yielding a stable macroporous network conducive to molecular diffusion and cellular trafficking.

Scanning electron microscopy (SEM) further confirmed these microstructural features (Figure 2G). The DGDA‐N hydrogel exhibited a compact morphology with narrowly distributed micropores (Figure 2H), whereas all emulsified hydrogels formed a hierarchical, honeycomb‐like architecture with extensive pore interconnection. Although incorporation of dopamine and Laponite led to a progressive reduction in pore size, the resulting pore dimensions in the DGDL hydrogel remained within a physiologically favorable range for nutrient transport and cell migration [53, 54]. Importantly, this controlled refinement of pore size balances permeability with mechanical stability, a critical requirement for sustaining pulp tissue under physiological pressure.

Elemental mapping by SEM‐EDS further verified the compositional homogeneity of the DGDL hydrogel (Figure 2I). Characteristic signals of Mg and Si from Laponite were uniformly co‐localized with organic elements (C and O), confirming the even dispersion of nanoclay throughout the polymer network. This homogeneous distribution ensures consistent mechanical reinforcement and uniform availability of bioactive ions, which is essential for delivering spatially consistent odontogenic cues to recruited dental pulp stem cells throughout the scaffold. In addition, comprehensive rheological profiling revealed that all hydrogel formulations exhibited shear‐thinning behavior (Figure S7). Emulsification markedly increased precursor viscosity relative to non‐porous formulations, reflecting the formation of a structured fluid. Dopamine functionalization further enhanced viscosity through catechol‐mediated hydrogen bonding, while Laponite incorporation produced the highest viscosity among all groups. Despite these increases, the DGDL precursor maintained excellent injectability, satisfying the practical requirements for minimally invasive pulp capping procedures.

### 2.3 Mechanical Characterization and Physicochemical Properties of DGDL Hydrogel

**FIGURE 3:**
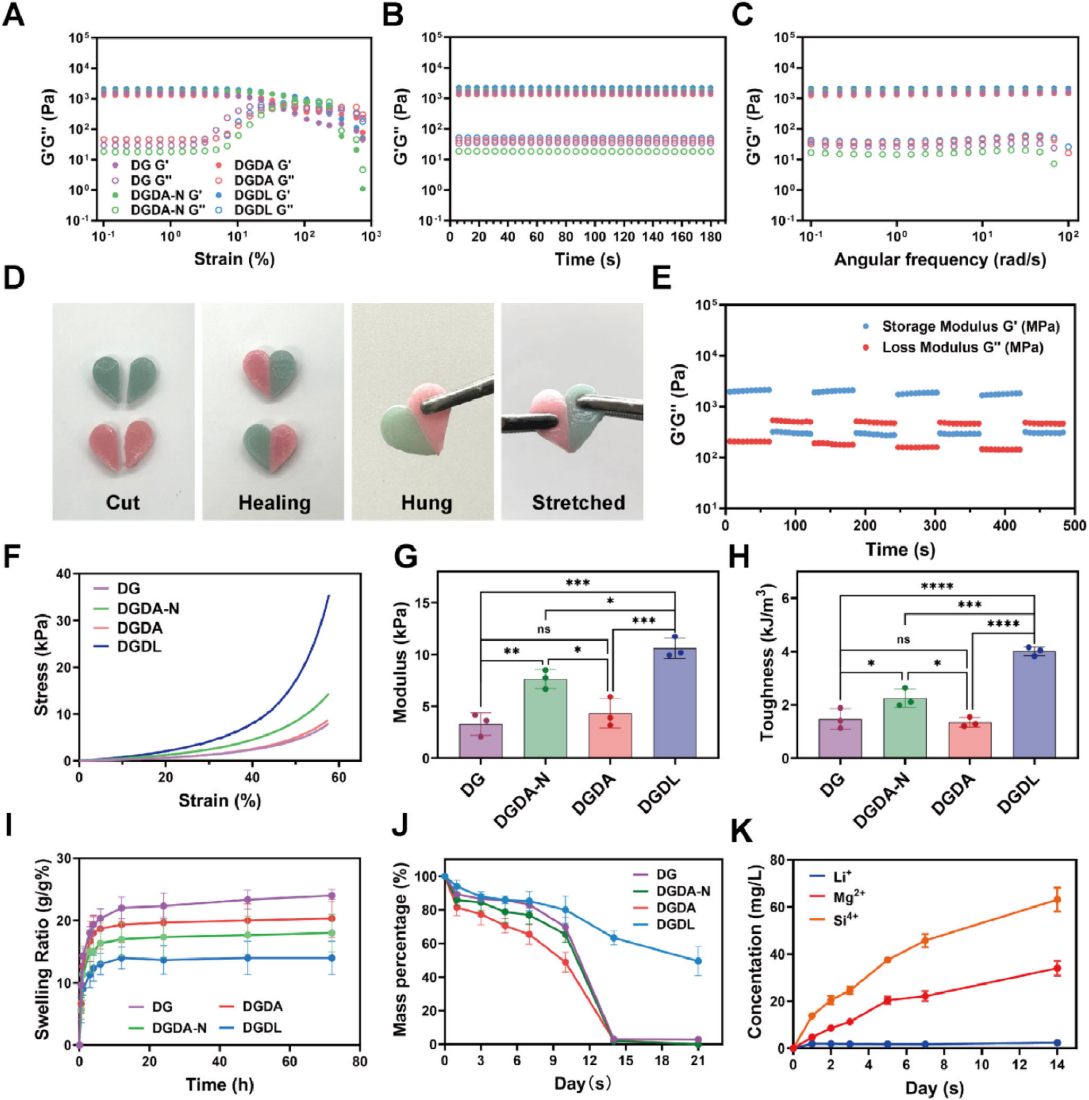
Mechanical characterization and physicochemical properties of DGDL hydrogel. (A–C) Rheological evaluation demonstrating viscoelastic stability via (A) strain, (B) time, and (C) frequency sweeps; (D, E) Assessment of self‐healing capability via (D) macroscopic stretching of healed samples and (E) continuous step‐strain alternative sweep demonstrating dynamic reversibility. (F–H) Assessment of compressive properties: (F) unconfined compression tests, along with quantified (G) compressive modulus and (H) toughness. (I–K) Evaluation of physicochemical properties: (I) swelling behavior, (J) resistance to enzymatic degradation, and (K) cumulative release of Mg^2+^, Li^+^ and Si^4+^ ions. Data represent mean ± SD (n = 3), *p< 0.05, **p < 0.01, ***p < 0.001.

Adequate mechanical integrity is a prerequisite for pulp capping materials to maintain structural stability under physiological conditions and intermittent masticatory loading [55]. Oscillatory rheological measurements were therefore performed to evaluate the viscoelastic behavior of the DGDL hydrogel. Strain sweep analysis demonstrated that all formulations exhibited solid‐like characteristics within the linear viscoelastic region, as evidenced by storage moduli (G') consistently exceeding loss moduli (G'') (Figure 3A). Time sweep tests further confirmed stable gelation behavior over the tested duration (Figure 3B), indicating the formation of mechanically resilient networks. Notably, incorporation of dopamine into the GelMA backbone resulted in a moderate reduction in modulus in the DGDA group relative to the DG control, which can be attributed to the UV‐shielding and radical‐scavenging properties of catechol moieties that partially interfere with photopolymerization efficiency. Importantly, this mechanical compromise was effectively counterbalanced by the introduction of Laponite nanoplates [45, 56]. The DGDL hydrogel exhibited the highest stiffness among all groups, exceeding even the dense DGDA‐N formulation. This observation confirms that Laponite serves as an efficient nanoreinforcement phase, compensating for dopamine‐induced attenuation and restoring mechanical robustness through physical crosslinking and stress transfer mechanisms. Frequency sweep analysis further corroborated the stability of the DGDL network, with G' remaining consistently elevated across the frequency range (Figure 3C), indicating restricted polymer chain mobility and enhanced resistance to dynamic deformation. Collectively, these rheological results demonstrate that the DGDL hydrogel achieves a critical balance between porosity and mechanical integrity, ensuring sufficient structural stability to sustain prolonged residence within the pulp chamber while preserving the open architecture necessary for microenvironmental regulation.

Beyond mechanical strength, the clinical success of vital pulp therapy critically depends on the handling characteristics and adaptability of the capping material within the confined pulp chamber. During clinical procedures, incremental placement and contouring are often required, and failure to achieve seamless integration between newly applied and existing material layers may introduce interfacial defects that compromise sealing efficacy. The incorporation of supramolecular DNA networks endows the DGDL hydrogel with intrinsic self‐healing capability mediated by reversible hydrogen bonding between complementary base pairs [57]. This dynamic reversibility enables autonomous structural repair without external stimuli, thereby facilitating seamless integration of fractured or incrementally added hydrogel segments.

Macroscopic self‐healing was directly visualized, as severed hydrogel pieces rapidly rejoined at the fracture interface, restoring structural continuity and tensile integrity (Figure 3D). Quantitative evaluation using continuous step‐strain rheological tests further confirmed this dynamic behavior (Figure 3E). Upon application of high strain (500%), the hydrogel network underwent reversible disruption, evidenced by a transient decrease in G'. Remarkably, immediate recovery of the original storage modulus was observed upon restoration to low strain (1%), demonstrating rapid network reassembly. This process remained stable over multiple rupture–healing cycles, reflecting excellent fatigue resistance and validating the “molecular zipper” mechanism conferred by DNA motifs. Such dynamic self‐healing behavior not only enhances clinical operability but also ensures the long‐term integrity of the pulp seal, which is essential for maintaining a stable microenvironment conducive to immune modulation and tissue regeneration.

To validate the material's ability to form a hermetic seal despite the inherent biomechanical mismatch between the hydrogel and dentin, we performed an interfacial evaluation using extracted human teeth (Figure S8). A standardized Class‐II cavity with pulp exposure model was prepared to mimic a clinical vital pulp therapy scenario (Figure S8A). Upon injection, the flowability and shear‐thinning behavior of the DGDL precursor allowed it to intimately adapt to the irregular dentinal walls. Following in situ photo‐crosslinking, the hydrogel rapidly transitioned into a stable, solid matrix that fully filled the defect (Figure S8B).

The SEM image of the hydrogel‐dentin interface demonstrated a tight, continuous integration (Figure S8C). Despite the dehydration process required for SEM preparation, no obvious interfacial gaps or micro‐fractures were observed at the junction. This exceptional marginal adaptation may synergistically drive by both physical and chemical mechanisms. The low‐viscosity precursor can effortlessly penetrate dentinal micro‐irregularities prior to gelation. The catechol groups grafted onto the GelMA‐DA backbone act as potent wet‐tissue adhesives, forming robust coordinate bonds with calcium ions exposed on the dentinal hydroxyapatite, alongside covalent crosslinks with the primary amines of dentinal collagen fibrils [58]. Concurrently, the extensive hydrogen‐bonding network facilitated by the supramolecular DNA motifs further reinforces this interfacial anchorage [59]. Consequently, the DGDL hydrogel forms a reliable, seamless integration that is crucial for preventing bacterial microleakage.

To quantitatively corroborate these morphological observations and evaluate the mechanical robustness of this biological seal, the adhesive properties of the DGDL hydrogel were systematically assessed. Ceramic plates were employed as testing substrates to simulate the rigid, inorganic, hydroxyapatite‐rich environment of dentin. The tensile testing results (Figure S9A) revealed a characteristic load‐displacement profile, wherein the DGDL hydrogel exhibited an increasing resistance up to a peak tensile strength of 7.6 kPa. This vertical adhesive behavior is primarily driven by the molecular engineering of the continuous polymer phase. The catechol groups function as the primary wet‐tissue adhesives, forming non‐covalent and potential coordination bonds with the inorganic ceramic surface, thereby effectively anchoring the hydrogel matrix [58]. Additionally, the innate adhesive nature of the incorporated macromolecular DNA, mediated through its extensive hydrogen‐bonding capabilities, further reinforces these interfacial interactions. Following the peak load, the displacement curve demonstrated a gradual decline rather than an abrupt drop. This suggests a cohesive‐dominant failure mode, indicating that the highly porous architecture and the dynamic coordination between the Laponite nanodisks and the polymer chains effectively dissipate mechanical energy during the pulling process.

To further mimic the clinical shear stresses encountered during the placement and condensation of restorative materials, the shear resistance of the hydrogel was validated through lap‐shear experiments. As depicted in Figure S9B, the DGDL hydrogel demonstrated a resilient linear elastic response under shear stress, culminating in a maximum lap‐shear strength of 15.5 kPa. The synergistic non‐covalent interactions between the Laponite disks and the DNA‐GelMA‐DA network generate abundant sacrificial bonds, which dramatically enhance interfacial stability and shear force dissipation. Collectively, these findings demonstrate that the biomimetic adhesive mechanisms of the DGDL hydrogel provide sufficient mechanical strength to withstand external operative stimuli. By maintaining strict interfacial stability, the hydrogel establishes a reliable, hermetic barrier that protects the pulp‐dentin complex and supports the long‐term success of subsequent tissue regeneration.

To further assess the mechanical suitability of the DGDL hydrogel under physiological conditions, unconfined compression tests were conducted. All hydrogel formulations exhibited non‐linear strain‐stiffening behavior characteristic of soft biological tissues (Figure 3F). Quantitative analysis indicated that the porous hydrogels without reinforcement (DG and DGDA) displayed lower compressive modulus and toughness compared to the dense DGDA‐N control (Figure 3G,H). Importantly, incorporation of Laponite significantly reinforced the network. The DGDL hydrogel exhibited the highest compressive modulus and toughness among all groups, highlighting the efficacy of nanoclay reinforcement in enhancing load‐bearing capacity while retaining porosity. This reinforced yet compliant mechanical profile enables the DGDL scaffold to withstand pulpal pressure and transient mechanical stresses without collapse or excessive deformation. Taken together, these mechanical and dynamic properties ensure that the DGDL hydrogel can maintain structural integrity, sealing performance and functional continuity throughout the regenerative process.

For successful vital pulp therapy, the capping scaffold must exhibit physicochemical properties that are temporally coordinated with tissue regeneration. An ideal material should maintain structural integrity during the early inflammatory and reparative phases, while gradually degrading to accommodate newly formed tissue without inducing secondary damage [59, 60, 61]. To evaluate this balance, the swelling behavior, enzymatic degradation and ion release kinetics of the hydrogels were systematically investigated. As shown in Figure 3I, all hydrogel formulations exhibited rapid initial water uptake followed by stabilization within 12 h, reflecting the establishment of hydration equilibrium. Notably, the DGDL hydrogel displayed a significantly reduced swelling ratio compared to the DG control. This restrained volumetric expansion is attributed to the incorporation of Laponite, which introduces additional physical crosslinking points that restrict polymer chain relaxation upon hydration. In the context of inflamed pulp tissue, where edema and elevated intra‐tissue pressure are common, such controlled swelling is particularly advantageous. By minimizing excessive expansion, the DGDL hydrogel reduces the risk of additional mechanical compression on the pulp, thereby mitigating pain, ischemia or secondary tissue necrosis.

Enzymatic degradation assays further revealed pronounced differences in structural persistence among the formulations (Figure 3J). Non‐reinforced hydrogels (DG, DGDA and DGDA‐N) underwent rapid proteolytic degradation, resulting in complete disintegration within 14 days. In contrast, the Laponite‐reinforced DGDL hydrogel exhibited markedly enhanced resistance to enzymatic erosion, retaining approximately 50% of its original mass after 21 days. This prolonged structural stability ensures sustained mechanical protection of the exposed pulp and preserves the porous framework necessary for immune modulation and endogenous stem cell activity during the critical phase of reparative dentin bridge formation.

Beyond providing mechanical support, the DGDL hydrogel functions as a dynamic reservoir for the sustained release of bioactive ions. Inductively coupled plasma–optical emission spectroscopy (ICP‐OES) analysis revealed continuous release of silicon, magnesium and lithium ions over a 14‐day period (Figure 3K). The release profiles suggest that the ionic liberation is closely coupled to gradual matrix erosion rather than being governed solely by rapid surface diffusion. Quantitatively, cumulative concentrations of Si, Mg and Li reached approximately 63, 34 and 2.5 mg/L, respectively. Such sustained ionic delivery is known to promote odontogenic differentiation and extracellular matrix mineralization, thereby contributing to the establishment of a pro‐regenerative microenvironment that supports reparative dentinogenesis [46, 62]. Collectively, these physicochemical characteristics demonstrate that the DGDL hydrogel provides not only spatial but also temporal control over the pulp healing process, maintaining a stable, bioactive niche during early inflammation while gradually transitioning to accommodate newly regenerated tissue.

### 2.4 In situ Cytocompatibility and Odontogenic Responsiveness of hDPSCs Induced by the DGDL Hydrogel

**FIGURE 4:**
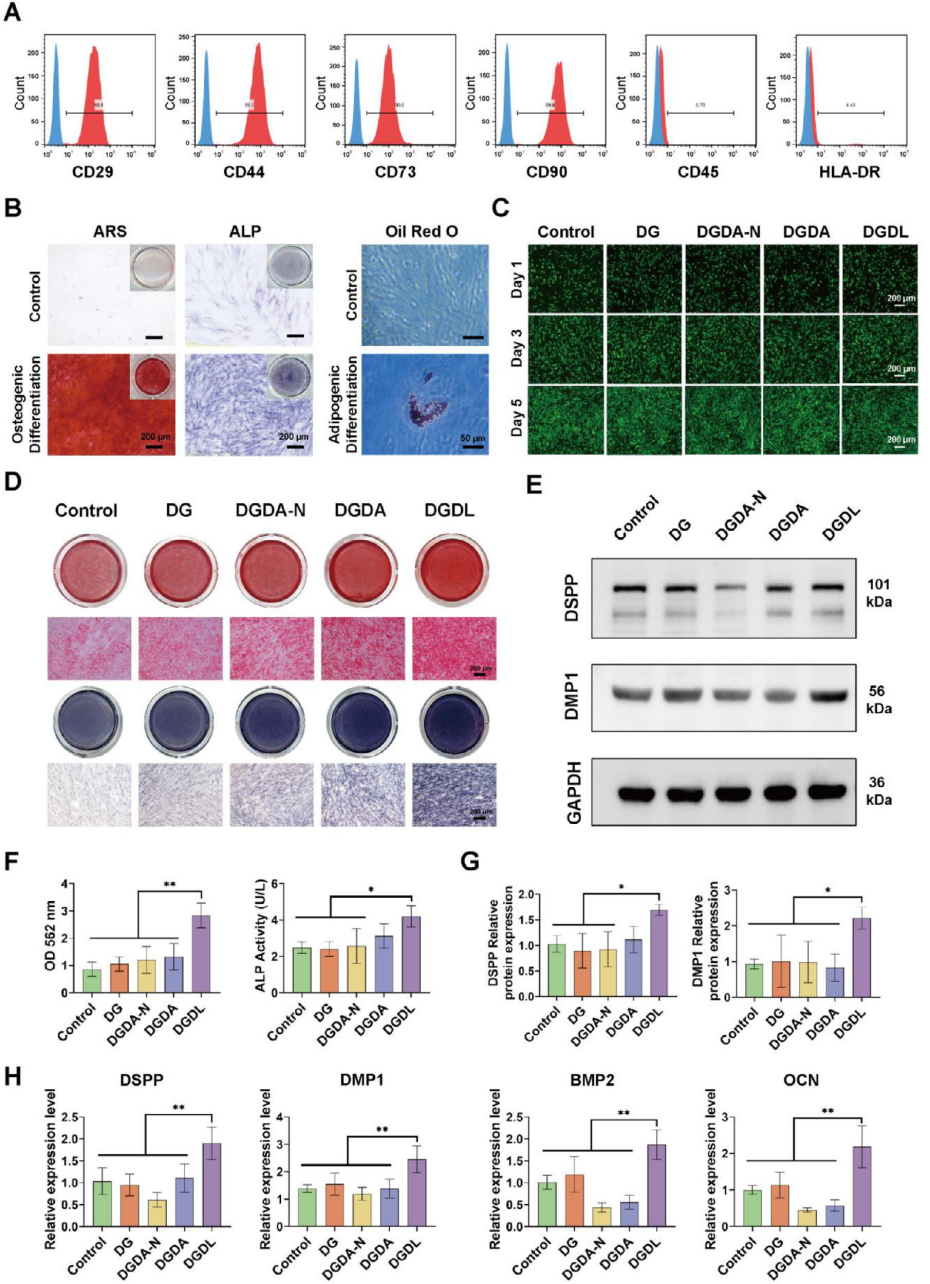
Odontogenic differentiation potential of hDPSCs in DGDL hydrogels. (A, B) Identification of hDPSCs: (A) Flow cytometry confirming stem cell surface marker expression; (B) Multilineage potential validation via odontogenic (ARS/ALP) and adipogenic (Oil Red O) staining. (C) Live/Dead staining assay demonstrating high biocompatibility and proliferation over 5 days (Scale bar: 200 µm). (D, F) Qualitative and quantitative analysis of mineralization (ARS) and ALP activity; (E, G) Western blot analysis of DSPP and DMP1 protein levels; RT‐qPCR profiling of key osteogenic genes (DSPP, DMP1, BMP2, OCN). (*p< 0.05, **p < 0.01).

Dental pulp stem cells (DPSCs) constitute the primary endogenous progenitor population responsible for reparative dentinogenesis and functional restoration of the pulp‐dentin complex [63]. Successful vital pulp therapy therefore critically depends on whether the implanted scaffold can provide a permissive microenvironment that supports DPSC survival, proliferation and lineage commitment under inflammatory conditions [63, 64]. Prior to evaluating the regenerative performance of the DGDL hydrogel, the biological identity and functional competence of the isolated human DPSCs (hDPSCs) were systematically confirmed. Flow cytometric analysis revealed that the isolated hDPSCs exhibited a typical mesenchymal stem cell immunophenotype, with high expression of CD29, CD44, CD73 and CD90 (>99%), and negligible expression of hematopoietic and immune markers CD45 and HLA‐DR (Figure 4A). In addition, multilineage differentiation assays verified their intrinsic multipotency, as evidenced by positive Alizarin Red S (ARS) and alkaline phosphatase (ALP) staining following odontogenic induction, and lipid droplet formation upon adipogenic differentiation (Figure 4B). These results confirmed that the isolated hDPSCs possess the requisite stemness and differentiation potential relevant to dentin–pulp regeneration.

The cytocompatibility of the DGDL hydrogel was subsequently assessed to determine whether its porous, dynamically crosslinked microenvironment supports hDPSC viability and proliferation. Live/Dead staining demonstrated predominantly viable cells with minimal apoptotic signals across all experimental groups throughout a 5‐day culture period (Figure 4C). Notably, hDPSCs cultured within the DGDL hydrogel displayed progressive cell proliferation and well‐spread morphologies, indicative of favorable cell–matrix interactions. These observations were further corroborated by quantitative CCK‐8 assays (Figure S10), which revealed sustained metabolic activity over time. Given the intended clinical application in direct contact with vascularized pulp tissue, hemocompatibility was also evaluated. All hydrogel formulations exhibited negligible hemolytic activity (Figure S11), confirming that the DGDL hydrogel does not induce adverse blood–material interactions. Collectively, these results demonstrate that the DGDL hydrogel establishes a cytocompatible and hemocompatible microenvironment that preserves hDPSC viability and functional competence, forming a necessary biological foundation for subsequent evaluations of odontogenic differentiation and regenerative performance.

The ultimate objective of bioactive pulp capping materials extends beyond maintaining cell viability, and instead lies in actively orchestrating odontogenic differentiation to enable functional reparative dentin formation [63, 65]. Building upon the favorable cytocompatibility and physicochemical characteristics of the DGDL hydrogel, we next evaluated whether its bioactive microenvironment could directly promote odontogenic commitment of hDPSCs at transcriptional, translational and phenotypic levels. As shown in Figure 4D,F, DPSCs cultured within the DGDL hydrogel exhibited significantly enhanced alkaline phosphatase (ALP) activity and mineral deposition, as evidenced by Alizarin Red S (ARS) staining, compared with all other groups. These phenotypic findings were further substantiated at the protein level. Western blot analysis revealed marked upregulation of the odontogenic markers dentin sialophosphoprotein (DSPP) and dentin matrix acidic phosphoprotein 1 (DMP1) in the DGDL group, with expression levels significantly exceeding those observed in non‐reinforced or non‐porous controls (Figure 4E,G). Consistently, RT‐qPCR analysis demonstrated that the DGDL hydrogel induced the highest mRNA expression of key odontogenic and mineralization‐associated genes, including DSPP, DMP1, BMP2 and osteocalcin (OCN) (Figure 4H). Together, these results indicate that the DGDL hydrogel establishes a microenvironment that robustly drives odontogenic differentiation of DPSCs rather than merely supporting baseline lineage commitment.

Mechanistically, the enhanced odontogenic response can be attributed to the sustained and coordinated release of bioactive ions from Laponite nanoplates embedded within the DGDL matrix during gradual scaffold degradation. Inductively coupled plasma analysis confirmed continuous release of lithium (Li^+^), magnesium (Mg^2+^) and silicon (Si^4+^), which collectively constitute a pro‐regenerative ionic milieu. Previous studies have demonstrated that Li^+^ activates Wnt/*β*‐catenin signaling through inhibition of glycogen synthase kinase 3 (GSK3), thereby modulating inflammation and promoting tissue regeneration [66] In parallel, Mg^2+^ has been shown to activate the ERK/BMP2/Smads signaling axis, driving DPSC odontogenic differentiation while suppressing pro‐inflammatory cytokines such as IL‐6 and TNF‐*α* [67, 68]. Meanwhile, Si^4+^ contributes to extracellular matrix stabilization and enhances mineral deposition during dentin formation [69]. These findings demonstrate that the DGDL hydrogel does not function as a passive differentiation scaffold, but rather as an active microenvironmental regulator that delivers sustained biochemical cues to synchronize inflammation resolution with odontogenic differentiation. While some previous literature suggests that dopamine can influence the viability and induce the osteo/odontogenic differentiation of stem cells from the apical papilla (SCAPs) in a dose‐ and time‐dependent manner [70], our findings showed that the robust and stable upregulation of differentiation markers only occurred upon the incorporation of Laponite. This coordinated signaling environment accelerates the transition of DPSCs from a reparative state to a mineralizing phenotype, thereby providing a mechanistic basis for effective reparative dentin bridge formation during vital pulp therapy.

### 2.5 Redox Homeostasis Restoration and Immunomodulation Enabled by the DGDL Hydrogel

**FIGURE 5:**
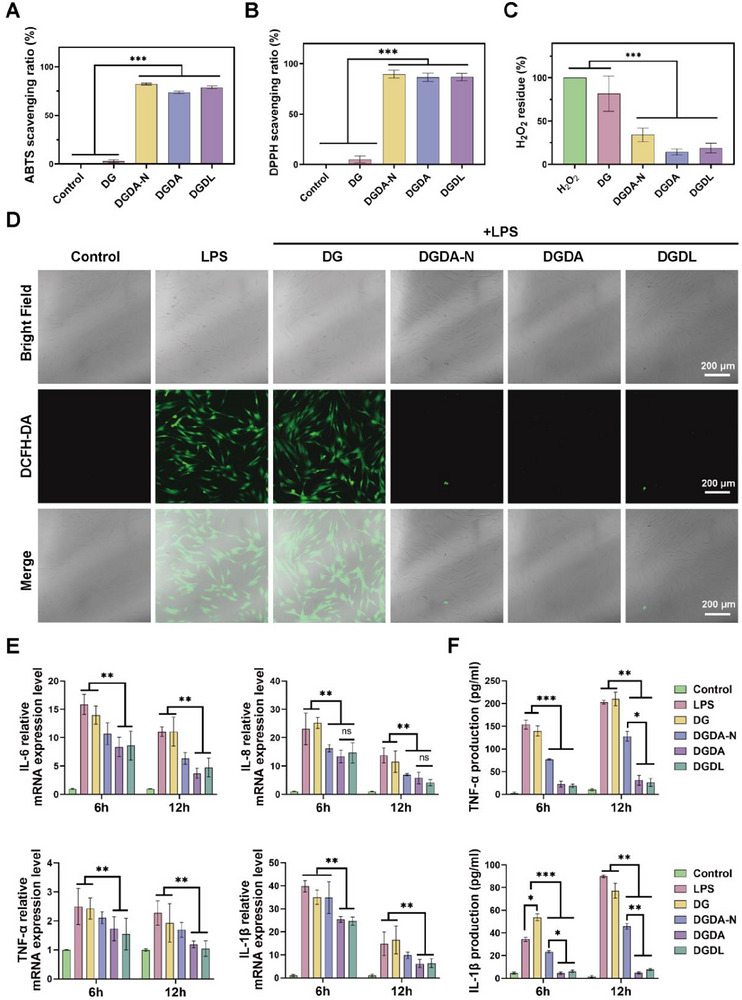
In vitro antioxidant and anti‐inflammatory efficacy of DGDL‐based hydrogels. (A–C) Evaluation of free radical scavenging capacity via (A) ABTS, (B) DPPH, and (C) H_2_O_2_ clearance assays. Catechol‐functionalized hydrogels (DGDA‐N, DGDA, DGDL) exhibited significantly enhanced antioxidant activity. (D) Representative fluorescence images of intracellular ROS levels in LPS‐stimulated hDPSCs, assessed by DCFH‐DA staining. Green fluorescence indicates ROS accumulation. (E) RT‐qPCR analysis of pro‐inflammatory cytokine gene expression (IL‐6, IL‐8, TNF‐*α*, IL‐1*β*) in hDPSCs at 6 and 12 h post‐LPS stimulation. (F) Secretion levels of TNF‐*α* and IL‐1*β* proteins measured by ELISA in hDPSCs after treatment with hydrogel extracts. Data are presented as mean ± SD (n = 3), *p< 0.05, **p < 0.01, ***p < 0.001.

Excessive accumulation of reactive oxygen species (ROS) is a hallmark of early pulpal injury and inflammation, acting as a critical upstream driver that amplifies inflammatory signaling and impairs the regenerative capacity of resident DPSCs [71]. Elevated ROS levels can induce DNA damage, disrupt cell cycle progression and compromise odontogenic differentiation, ultimately trapping the pulp tissue in a non‐regenerative inflammatory state [72]. Notably, conventional pulp‐capping materials lack intrinsic antioxidant activity and therefore fail to actively reverse this hostile redox microenvironment upon application. Restoring redox homeostasis is thus a prerequisite for effective inflammation resolution and active pulp preservation. In DGDL hydrogel, dopamine‐derived catechol moieties were introduced as intrinsic redox‐regulatory motifs. Catechol groups are well‐established antioxidants capable of neutralizing free radicals through electron donation and hydrogen atom transfer mechanisms [73]. We therefore hypothesized that GelMA–DA would function as an endogenous antioxidant component to actively scavenge excessive ROS and re‐establish a favorable redox balance within the inflamed pulp tissue. The antioxidant capacity of the hydrogels was first evaluated using ABTS, DPPH and H_2_O_2_ scavenging assays. As shown in Figure 5A–C, the unmodified DG hydrogel exhibited negligible free radical scavenging activity. Meanwhile, all dopamine‐functionalized hydrogels (DGDA‐N, DGDA and DGDL) demonstrated significantly enhanced antioxidant efficacy, confirming that catechol incorporation endowed the materials with robust ROS‐neutralizing capability.

To assess whether this antioxidant activity facilitated effective intracellular redox regulation, LPS‐stimulated hDPSCs were employed to model an oxidative stress–dominated inflammatory microenvironment. Intracellular ROS levels were visualized using the DCFH‐DA probe. As shown in Figure 5D, cells exposed to LPS or cultured with the DG hydrogel exhibited intense green fluorescence, indicative of severe intracellular ROS accumulation. In contrast, LPS‐stimulated cells treated with dopamine‐functionalized hydrogels (DGDA‐N, DGDA and DGDL) displayed markedly attenuated fluorescence intensity, with ROS levels restored to near‐physiological baselines comparable to untreated controls. These results demonstrate that GelMA‐DA–containing hydrogels can effectively mitigate intracellular oxidative stress in the inflamed DPSCs.

Given the well‐established crosstalk between oxidative stress and inflammatory signaling, we next investigated whether the restoration of redox balance could be translated into downstream immunomodulatory effects [74]. Quantitative RT‐qPCR analysis revealed significant downregulation of key pro‐inflammatory genes, including IL‐6, IL‐8, TNF‐*α* and IL‐1*β*, in LPS‐stimulated hDPSCs treated with dopamine‐functionalized hydrogels relative to the DG control at both 6 and 12 h (Figure 5E). Consistently, ELISA measurements confirmed substantially reduced secretion of TNF‐*α* and IL‐1*β* cytokines in these groups (Figure 5F). These findings indicate that effective ROS scavenging by the hydrogel attenuates oxidative stress–driven inflammatory cascades, thereby suppressing pro‐inflammatory cytokine production at both transcriptional and protein levels. These results demonstrate that the DGDL hydrogel actively restores redox homeostasis and reprograms the inflammatory microenvironment at an early stage, rather than passively tolerating oxidative damage. This redox‐driven immunomodulation establishes a permissive biological niche that is essential for subsequent endogenous stem cell recruitment and odontogenic differentiation, thereby serving as the initiating event in active pulp preservation.

### 2.6 Redox‐Driven Immunomodulation and Macrophage Phenotypic Reprogramming

**FIGURE 6:**
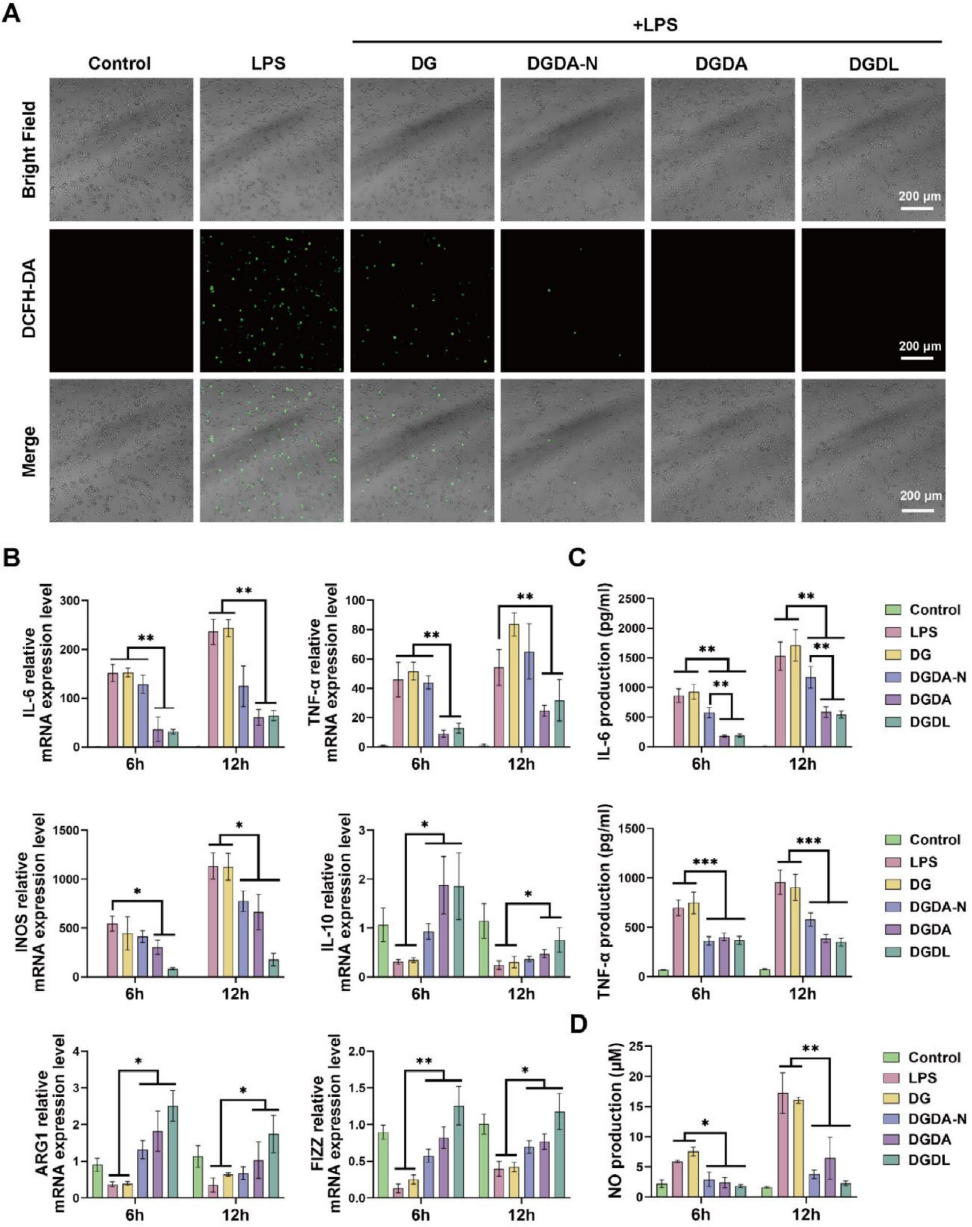
Immunomodulatory effects of DGDL hydrogels on macrophage polarization. (A) Representative fluorescence images depicting intracellular ROS levels in LPS‐stimulated RAW 264.7 macrophages, assessed via DCFH‐DA staining. Green fluorescence corresponds to ROS intensity. (B–D) Quantitative evaluation of macrophage phenotype polarization following treatment with hydrogel extracts: (B) RT‐qPCR analysis of M1‐associated (IL‐6, TNF‐*α*, iNOS) and M2‐associated (IL‐10, ARG1, FIZZ1) gene expression. (C) ELISA‐based quantification of secreted IL‐6 and TNF‐*α* proteins. (D) Measurement of nitric oxide (NO) production in culture supernatants. Data are expressed as mean ± SD (n = 3), *p< 0.05, **p < 0.01, ***p < 0.001.

Beyond progenitor cells, the immune microenvironment—governed primarily by macrophages—is also pivotal in determining the success of pulp capping therapy [75]. During pulpitis, infiltrating macrophages are rapidly activated and preferentially polarize toward a pro‐inflammatory M1 phenotype, accompanied by a pronounced respiratory burst and excessive production of ROS [76]. This sustained oxidative stress amplifies inflammatory signaling, disrupts tissue homeostasis, and ultimately predisposes the pulp to irreversible damage or necrosis [76, 77]. Accordingly, effective pulp preservation requires not only suppression of inflammatory mediators, but also active reprogramming of macrophage phenotype toward a pro‐regenerative state [78]. We therefore examined whether the antioxidant capability of the DGDL hydrogel could mitigate oxidative stress in activated macrophages. Intracellular ROS levels were visualized in LPS‐stimulated RAW 264.7 macrophages using DCFH‐DA staining (Figure 6A). While macrophages exposed to the DG hydrogel or LPS alone exhibited intense fluorescence indicative of excessive ROS accumulation, treatment with dopamine‐functionalized hydrogels markedly attenuated intracellular ROS levels. This effect confirms that catechol moieties embedded within the GelMA‐DA network serve as effective redox regulators in immune cells, consistent with the antioxidant behavior observed in hDPSCs.

Given the complex interplay between redox status and macrophage phenotype, we next investigated whether restoration of oxidative balance could be translated into functional immunomodulation. Under LPS stimulation, macrophages exhibited strong upregulation of M1‐associated markers, including inducible nitric oxide synthase (iNOS), IL‐6 and TNF‐*α*, accompanied by elevated nitric oxide (NO) production (Figure 6B,D). Exposure to dopamine‐functionalized hydrogels significantly suppressed these pro‐inflammatory markers at both transcriptional and functional levels. Notably, the DGDL group exerted the most pronounced inhibitory effect, reducing iNOS and IL‐6 mRNA expression by more than 70%, alongside a substantial decrease in NO production.

Concurrently, expression of M2‐associated markers was markedly enhanced. While macrophages treated with DG hydrogel or LPS alone showed negligible expression of the anti‐inflammatory and reparative markers arginase‐1 (ARG1), resistin‐like molecule alpha (FIZZ1), and IL‐10, dopamine‐functionalized hydrogels induced significant upregulation of these genes, with the DGDL group again demonstrating the strongest effect (Figure 6B). ELISA analysis further corroborated these findings, revealing significantly reduced secretion of IL‐6 and TNF‐*α* in DGDL‐treated macrophages (Figure 6C). Together, these results indicate a clear phenotypic shift from a destructive M1 state toward a pro‐regenerative M2 phenotype. Importantly, this immunomodulatory effect extends beyond passive suppression of inflammation. By restoring redox homeostasis, the DGDL hydrogel actively reprograms macrophage behavior, reshaping the immune microenvironment toward one that supports tissue repair rather than perpetuating damage. Such redox‐driven macrophage polarization provides a critical immunological foundation for endogenous stem cell recruitment, odontogenic differentiation and ultimately functional pulp–dentin regeneration.

### 2.7 Screening and Identification of High‐Affinity Aptamers for DPSCs and Cell Recruitment

**FIGURE 7:**
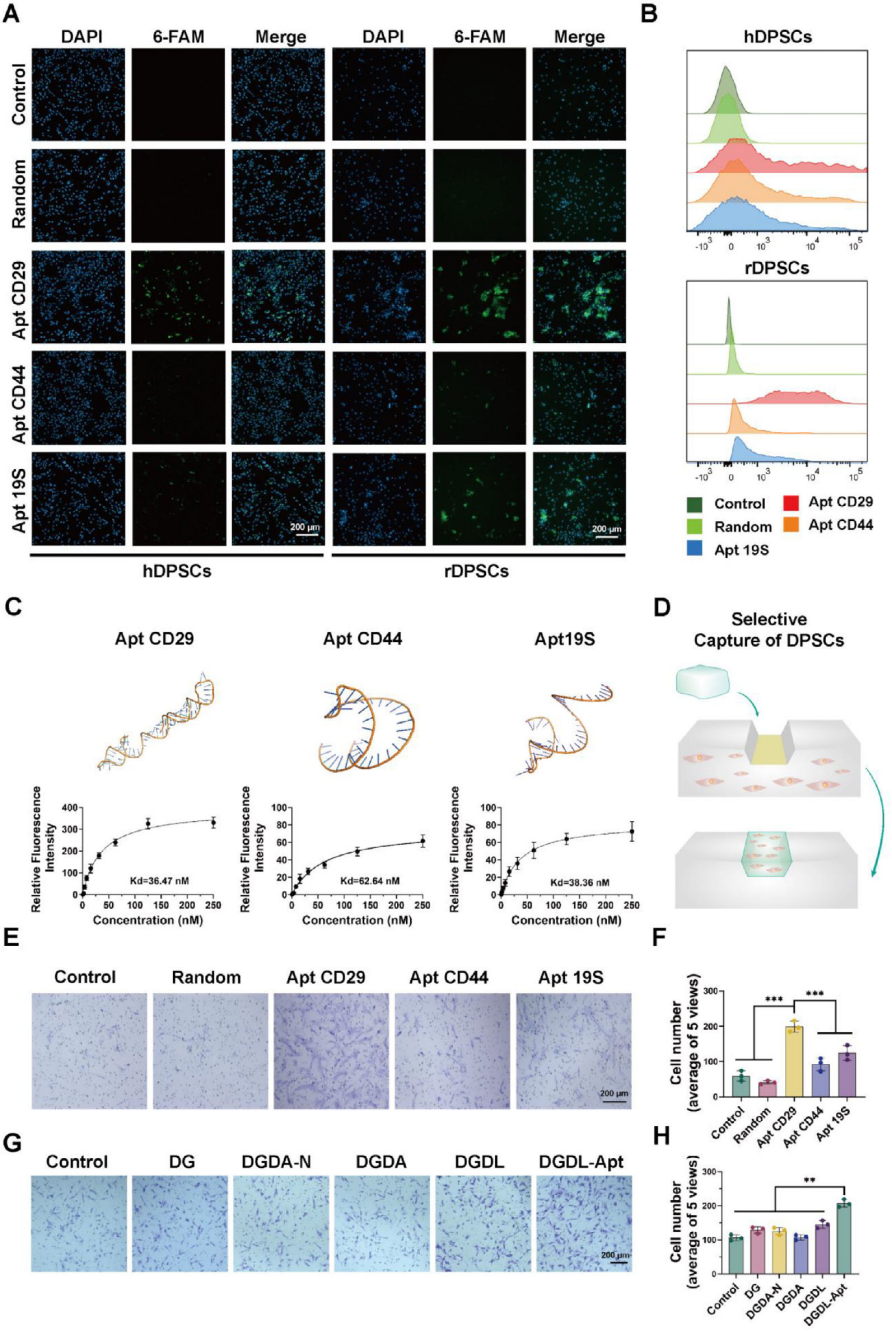
Screening, validation and functional application of DPSC‐targeting aptamers. (A–D) Affinity screening and structural characterization of candidate aptamers. (A) Representative fluorescence images showing specific binding of FAM‐labeled aptamers to human (hDPSCs) and rat (rDPSCs) dental pulp stem cells. (B) Flow cytometry histograms comparing the binding intensities of Apt‐CD29, Apt‐CD44 and Apt‐19S to DPSCs. (C) Predicted tertiary structures, saturation binding curves and corresponding equilibrium dissociation constants (K_d_) of the three aptamers, demonstrating that Apt‐CD29 exhibits the highest binding affinity. (D) Schematic illustration of aptamer‐mediated in situ DPSC recruitment. The CD29‐targeting aptamer acts as a molecular anchor to specifically capture and retain endogenous DPSCs within the hydrogel scaffold. (E–H) Functional validation of aptamer‐mediated cell recruitment. (E, F) Transwell migration assay assessing the chemotactic effect of different aptamers on hDPSCs: (E) representative images of migrated cells and (F) corresponding quantitative analysis. (G, H) Recruitment efficiency of the aptamer‐functionalized hydrogel (DGDL‐Apt) compared to the non‐functionalized control: (G) representative images and (H) quantitative cell counts on the migration membrane. Data are presented as mean ± SD (n = 3), Statistical significance: **p < 0.01, ***p < 0.001.

While restoration of redox balance and immunological homeostasis establishes a permissive microenvironment for pulp regeneration, successful tissue repair ultimately requires the timely presence of competent endogenous progenitor cells. In inflamed pulp tissue, however, the local pool of functional DPSCs is often insufficient or spatially dispersed, limiting the regenerative response even after inflammation resolution. We therefore sought to introduce a molecular targeting strategy capable of actively recruiting endogenous DPSCs to the injury site. Aptamers are single‐stranded oligonucleotides that adopt defined 3D conformations, enabling high‐affinity and highly specific interactions with cell‐surface receptors [79]. Owing to these properties, aptamers have emerged as powerful tools for cell‐selective recognition and in situ cell recruitment [80, 81]. To identify an optimal aptamer for DPSC targeting, three candidate sequences were systematically screened, including aptamers recognizing mesenchymal stem cell surface markers CD29 and CD44, as well as Aptamer 19S, which has been reported totarget pluripotent stem cells [82, 83, 84]. Specific binding of candidate aptamers was first evaluated using both human and rat DPSCs. Fluorescence imaging revealed that Apt‐CD29 exhibited markedly stronger binding affinity to hDPSCs and rDPSCs compared to Apt‐CD44 and Apt‐19S (Figure 7A). Flow cytometry analysis further confirmed this observation, showing a pronounced rightward shift in fluorescence intensity for Apt‐CD29 (Figure 7B). Structural modeling demonstrated that all candidate aptamers folded into stable tertiary architectures featuring helical domains and loop motifs required for molecular recognition (Figure 7C). Quantitative saturation binding analysis revealed that Apt‐CD29 achieved the highest maximal binding capacity (B_max_) and the lowest equilibrium dissociation constant (K_d_), indicating superior affinity and capture potential (Figure 7C). Although Apt‐19S exhibited a comparable K_d_ value, its substantially lower B_max_ suggested limited overall recruitment efficiency. Based on these results, Apt‐CD29 was selected as the optimal targeting ligand for subsequent endogenous DPSC recruitment (Figure 7D).

To validate the functional recruitment capability of the selected aptamer, Transwell migration assays were performed. As illustrated in Figure 7E,F, Apt‐CD29 induced significantly enhanced transmembrane migration of hDPSCs compared to Apt‐CD44 and Apt‐19S, confirming its superior cell‐attracting capability. To further confirm the selectivity and specific capture efficiency of the incorporated Apt‐CD29 for stem cells, an in vitro capture assay was conducted using hDPSCs. To allow for precise quantitative analysis, non‐emulsified flat hydrogels were employed. As illustrated in Figure S12A, robust cell capture was observed exclusively on the Apt‐CD29 functionalized hydrogels at 6 h post‐seeding. A substantial number of hDPSCs firmly adhered to these surfaces and exhibited extensive, well‐spread cellular morphologies. The unfunctionalized control hydrogels, as well as those conjugated with Apt‐CD44 and Apt‐19S however, exhibited minimal cell adhesion, with very few cells remaining on the surfaces following the washing steps. Quantitative analysis of the adherent cells (Figure S12B) further corroborated these observations, demonstrating a significantly superior capture efficiency in the Apt‐CD29 group compared to all controls.

The 6‐h observation window demonstrates that the initial cell recruitment is driven specifically by the rapid recognition between the aptamer and the CD29 receptors on the hDPSCs, effectively outpacing the non‐specific binding naturally mediated by the GelMA RGD motifs. These results validate the high selectivity and specific capture capability of the Apt‐CD29 sequence, providing a strong rationale for its incorporation into the DGDL matrix to actively recruit endogenous DPSCs.

Importantly, when Apt‐CD29 was immobilized within the DGDL hydrogel matrix (DGDL‐Apt), the scaffold exhibited a markedly increased ability to recruit DPSCs compared to hydrogels lacking aptamer functionalization (Figure 7G,H). These findings demonstrate that Apt‐CD29 retains its molecular recognition function after integration into the hydrogel network and can serve as a stable bio‐instructive cue for in situ stem cell capture.

To directly visualize and further substantiate this localized recruitment dynamic in a spatially defined context, a macroscopic in vitro cell recruitment assay was performed. DGDL and DGDL‐Apt hydrogels were patterned in the center of culture wells, and hDPSCs were seeded in the surrounding periphery to mimic the physiological migration of endogenous stem cells toward the central biomaterial implant. As illustrated in Figure S13, temporal monitoring of the hydrogel‐cell interfaces revealed distinct behavioral differences. At 12 h post‐seeding, cells in both groups had successfully attached and spread, with a slight directional migration trend observed toward the DGDL‐Apt hydrogel.

However, by 24 h, a prominent morphological divergence was observed. In the DGDL‐Apt group, a substantial density of hDPSCs had actively migrated toward the hydrogel, accumulating densely right at the material boundary. Conversely, while cells in the unfunctionalized DGDL group exhibited a mild tendency to migrate toward the scaffold, this non‐specific recruitment was notably limited. The cells surrounding the DGDL gel remained relatively sparse, lacking the targeted, dense accumulation clearly evident in the DGDL‐Apt group. These findings confirm that the incorporation of the CD29‐targeting aptamer endows the hydrogel with a robust capacity to actively attract and recruit surrounding stem cells, effectively bridging the spatial gap between the tissue and the scaffold.

Critically, effective cell recruitment must not compromise stem cell phenotype or functional potential. Analysis of pluripotency‐associated gene expression (Nanog, Sox2, Klf4, and Pou5f1) revealed no significant differences among aptamer‐treated groups (Figure S14), indicating preservation of stemness characteristics. Cell viability remained comparable to untreated controls across a range of aptamer concentrations (Figure S15). Furthermore, mineralized nodule formation and ALP activity assays confirmed that aptamer binding did not impair odontogenic differentiation capacity of DPSCs (Figure S16). These results collectively indicate that Apt‐CD29 functions as a molecular anchoring interface rather than a differentiation‐modulating signal. Taken together, aptamer‐mediated DPSC recruitment provides a critical biological bridge that links immune–redox microenvironmental reprogramming to effective tissue regeneration. By actively concentrating endogenous progenitor cells at the injury site, the DGDL‐Apt hydrogel ensures that the reprogrammed, pro‐regenerative microenvironment effectively drives functional reparative dentin formation during vital pulp therapy.

Additionally, the target‐binding affinity of the incorporated aptamers is not compromised by the hybridization with macromolecular DNA. Because aptamers exhibit microsecond‐scale intramolecular folding kinetics, they can rapidly form stable conformations that thermodynamically outcompete intermolecular hybridization with the DNA network. Consistent with its widespread application as a blocking agent in SELEX protocols, the DNA within the hydrogel matrix preferentially functions to mask non‐specific protein adsorption sites [84, 85]. This effectively reduces background interference and preserves the structural integrity and specific hDPSC‐targeting capability of the CD29 aptamers within the complex pulpal microenvironment.

### 2.8 In Vivo Evaluation of Active Pulp Preservation and Reparative Dentinogenesis in a Rat Pulpitis Model

**FIGURE 8:**
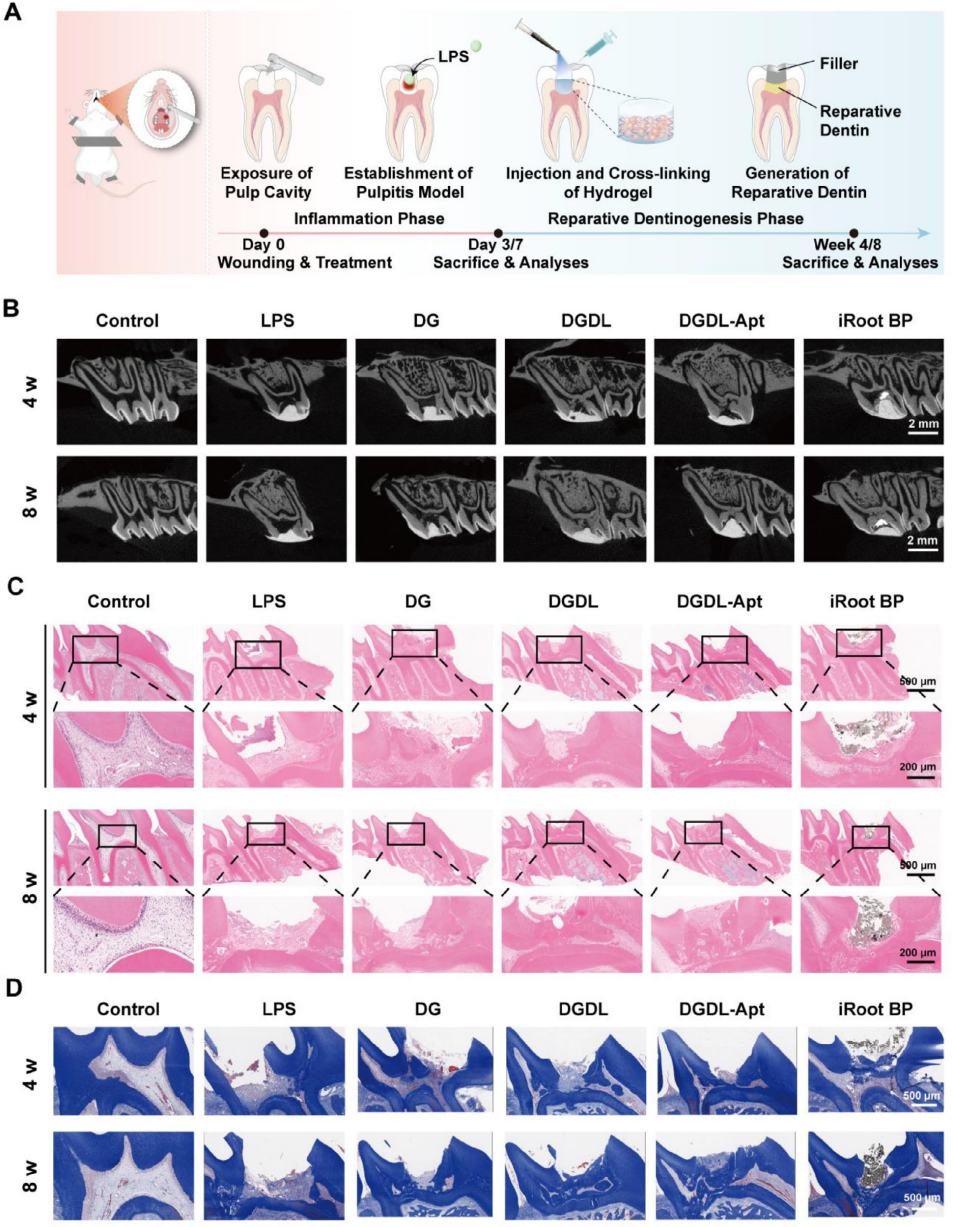
In vivo evaluation of reparative dentinogenesis in a rat pulpitis model. (A) Schematic illustration of the pulpitis rat model establishment and experimental timeline, including mechanical pulp exposure, LPS‐induced inflammation, hydrogel application and observation endpoints. (B) Representative Micro‐CT cross‐sectional images of maxillary molars at 4 and 8 weeks post‐treatment, showing reparative dentin formation within the pulp chamber. (C, D) Histological analysis of pulp‐dentin complex regeneration via (C) hematoxylin and eosin (H&E) staining and (D) Masson's trichrome staining.

To validate whether the immune–redox microenvironment reprogramming and targeted endogenous stem cell recruitment observed in vitro could be translated into functional tissue regeneration, a rat model of pulpitis was established [14] (Figure 8A). Following mechanical exposure of the pulp chamber, acute inflammation was induced by localized LPS stimulation. The exposed pulps were subsequently treated with different hydrogels (DG, DGDL or DGDL‐Apt) or the clinically used calcium silicate–based material iRoot BP, followed by in situ photopolymerization to achieve immediate hermetic sealing. Resin restoration was applied thereafter to prevent marginal microleakage and secondary infection.

Prior to evaluating regenerative outcomes, systemic biosafety was assessed. Histological examination of major organs, including the heart, liver, spleen, lung and kidney, at 2 months post‐treatment revealed no discernible pathological alterations across all groups (Figure S17), indicating that the hydrogel formulations exhibit favorable in vivo biocompatibility for pulp capping applications. Micro‐computed tomography (micro‐CT) was employed to longitudinally assess hard tissue regeneration within the pulp exposure site (Figure 8B). At 4 weeks, persistent radiolucency was observed in the LPS and DG groups, indicative of insufficient mineralized tissue formation. In contrast, both DGDL and DGDL‐Apt groups exhibited early‐stage deposition of mineralized tissue within the defect region. By 8 weeks, a continuous and well‐defined radiopaque barrier was clearly evident in the Laponite‐containing groups, with the DGDL‐Apt group showing the most pronounced dentin bridge formation. Notably, the morphology and continuity of the newly formed barrier in the DGDL‐Apt group closely resembled those observed in the iRoot BP group.

To quantitatively substantiate these radiographic findings, the quality of reparative dentin formation was semi‐quantitatively evaluated based on continuity, morphology and thickness (Tables 1, 2, 3, Supporting Information) [86]. At 4 weeks, the LPS and DG groups predominantly received the highest scores, confirming failure of effective hard tissue regeneration. In contrast, both DGDL and DGDL‐Apt groups demonstrated significantly lower scores, reflecting early regenerative responses. By 8 weeks, the DGDL‐Apt group exhibited the lowest scores across all parameters, surpassing the non‐aptamer DGDL group and approaching the performance of the clinical gold standard.

**TABLE 1:** Observation Result of Dentinal Bridge Continuity.

Capping Material	4 weeks				*p*(*)	8 weeks				*p*(*)
	Score 1	Score 2	Score 3	Score 4	< 0.05	Score 1	Score 2	Score 3	Score 4	< 0.05
Control	—	—	—	—		—	—	—	—	
LPS	0	0	0	6		0	0	0	6	
DG	0	0	1	5		0	0	1	5	
DGDL	0	0	5	1		3	2	1	0	
DGDL‐Apt	0	1	4	1		4	2	0	0	
iRoot BP	0	0	5	1		3	3	0	0	

Desc: Score 1: the dentinal bridge is completely formed; Score 2: dentinal bridge has formed covering more than half of the exposed area; Score 3: dentinal bridge initiation has been formed which has not covered half of the exposure area; Score 4: no dentinal bridge. (*) using Kruskal Wallis test with *α* = 0.05.

**TABLE 2:** Observation Result of Dentinal Bridge Morphology.

Capping Material	4 weeks				*p*(*)	8 weeks				*p*(*)
	Score 1	Score 2	Score 3	Score 4	< 0.05	Score 1	Score 2	Score 3	Score 4	< 0.05
Control	—	—	—	—		—	—	—	—	
LPS	0	0	0	6		0	0	0	6	
DG	0	0	1	5		0	0	1	5	
DGDL	0	0	5	1		3	2	1	0	
DGDL‐Apt	0	1	4	1		4	2	0	0	
iRoot BP	0	0	5	1		3	2	1	0	

Desc: Score 1: dentin was completely formed; Score 2: only irregular hard tissue deposition is formed; Score 3: only thin layer of hard tissue deposition is formed; Score 4: no hard tissue deposition. (*) using Kruskal Wallis test with *α* = 0.05.

**TABLE 3:** Observation Result of Dentinal Bridge Thickness.

Capping Material	4 weeks				*p*(*)	8 weeks				*p*(*)
	Score 1	Score 2	Score 3	Score 4	< 0.05	Score 1	Score 2	Score 3	Score 4	< 0.05
Control	—	—	—	—		—	—	—	—	
LPS	0	0	0	6		0	0	0	6	
DG	0	0	0	6		0	0	5	1	
DGDL	0	2	4	1		3	2	1	0	
DGDL‐Apt	0	3	3	0		4	2	0	0	
iRoot BP	0	0	3	3		3	3	0	0	

Desc: Score 1: >0.25mm; Score 2: 0.1‐0.25mm; Score 3: <0.1mm; Score 4: no dentinal bridge. (*) using Kruskal Wallis test with *α* = 0.05.

Hematoxylin and eosin (H&E) staining at 4 weeks revealed persistent inflammatory cell infiltration and absence of organized calcified tissue in the LPS and DG groups (Figure 8C). DGDL‐based treatments facilitated a marked reduction in defect areas. Notably, the DGDL‐Apt group demonstrated superior defect closure compared to the DGDL group, with a newly deposited matrix partially occluding the exposure site and presenting a well‐defined boundary from the primary dentin. By 8 weeks, only sparse and disorganized calcifications were observed in the LPS and DG groups, whereas the DGDL‐Apt group formed a thick, continuous reparative dentin bridge that was morphologically indistinguishable from that generated by iRoot BP. The maturity and organization of the regenerated extracellular matrix were further evaluated by Masson's trichrome staining (Figure 8D). Consistent with the H&E results, the DGDL‐Apt group displayed densely packed, well‐organized collagen bundles constituting the mineralized dentin bridge, indicative of advanced matrix maturation. In contrast, the LPS and DG control groups exhibited loose, disorganized fibrous tissue with limited collagen deposition. Importantly, the structural organization and collagen density achieved by the DGDL‐Apt hydrogel were comparable to those observed in the iRoot BP group. The in vivo findings demonstrate that the DGDL‐Apt hydrogel enables effective resolution of inflammation and promotes high‐quality reparative dentinogenesis in a pulpitis rat model.

### 2.9 Early‐Stage In Vivo Immunomodulation and Targeted Endogenous Stem Cell Recruitment

**FIGURE 9:**
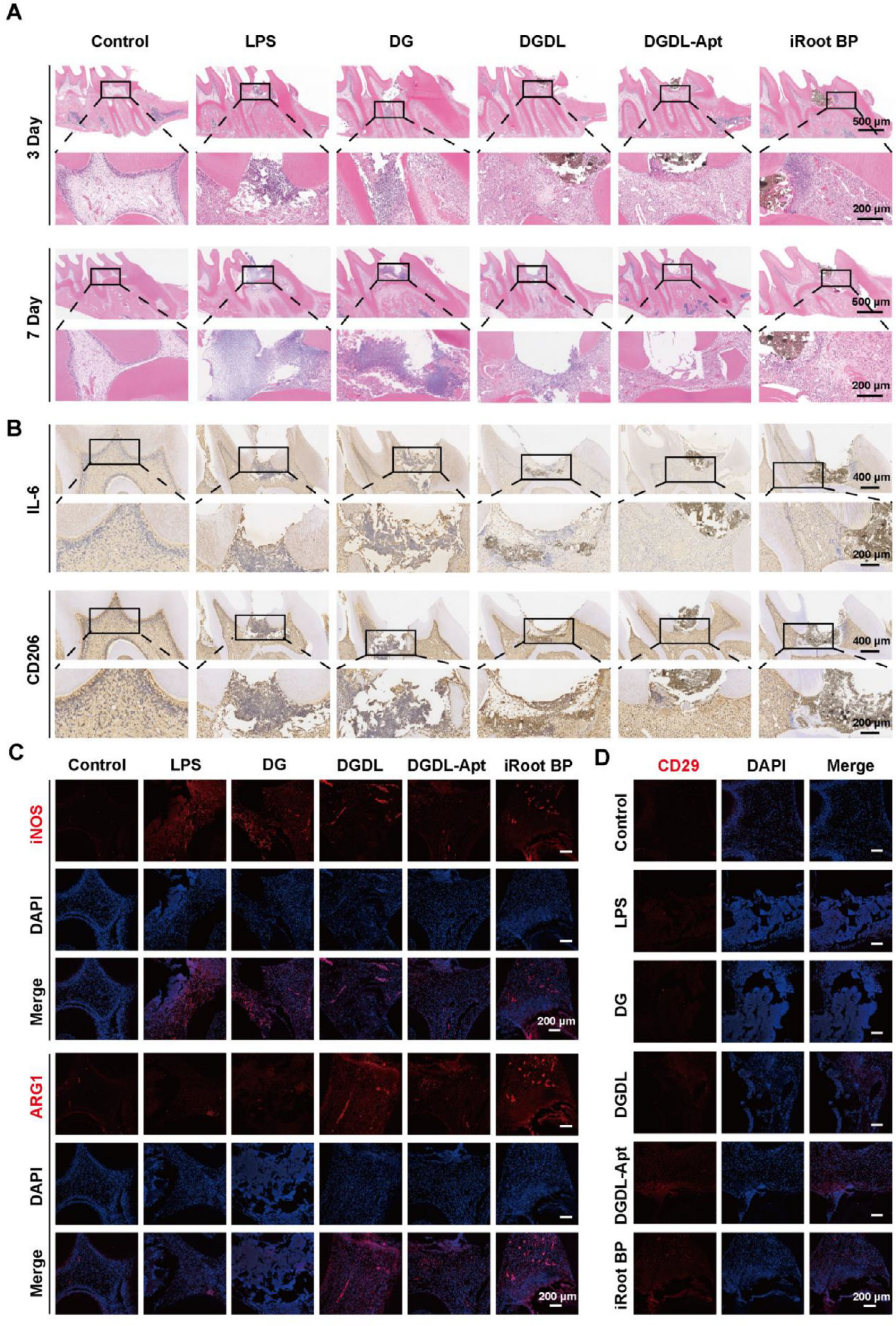
Assessment of early inflammatory resolution and in situ stem cell recruitment mediated by DGDL‐Apt hydrogel. (A) Representative hematoxylin and eosin (H&E)‐stained sections of rat dental pulp at 3 and 7 days post‐treatment. Inflammatory cell infiltration and tissue architecture are shown at low and high magnifications. (B) Immunohistochemical detection of inflammatory marker IL‐6 (pro‑inflammatory) and macrophage marker CD206 (M2‑associated) in pulp tissues at day 3. (C) Immunofluorescence staining of M1 marker iNOS and M2 marker ARG1 in pulp tissues at day 3. Nuclei are counterstained with DAPI (blue). (D) Immunofluorescence staining of CD29, a surface marker of DPSCs, in pulp tissues at day 3, showing recruitment of endogenous stem cells toward the hydrogel–pulp interface. Nuclei stained with DAPI (blue).

To elucidate the early biological events underlying the regenerative outcomes observed at later stages, the acute inflammatory response and in situ stem cell recruitment were systematically evaluated at 3 and 7 days following hydrogel implantation. These early time points are critical, as uncontrolled inflammation during this phase often predetermines failure of vital pulp therapy. Histological examination by H&E staining revealed pronounced differences among treatment groups at day 3 post‐operation (Figure 9A). The LPS and DG groups exhibited severe pathological alterations, including extensive hyperemia, interstitial edema and dense infiltration of inflammatory cells dominated by neutrophils, indicative of an uncontrolled acute inflammatory cascade. In contrast, pulp tissues treated with DGDL and DGDL‐Apt hydrogels maintained largely preserved tissue architecture with markedly reduced inflammatory cell infiltration. By day 7, these differences became more pronounced. While the LPS and DG groups continued to display diffuse and persistent inflammation, the inflammatory response in DGDL‐based groups was substantially attenuated and confined, consistent with the initiation of tissue repair processes.

To further define the immunological basis of this early inflammation resolution, macrophage phenotypes were assessed by immunohistochemical and immunofluorescence analyses. At day 3, strong expression of pro‐inflammatory markers IL‐6 and iNOS was observed in the LPS and DG groups, reflecting a dominant M1 macrophage phenotype (Figure 9B,C). In contrast, DGDL and DGDL‐Apt treatments markedly suppressed M1‐associated signals while simultaneously enhancing expression of the reparative M2 markers CD206 and ARG1. The corresponding quantitative analysis of iNOS and ARG1 immunofluorescence intensities is presented in Figure S19A,B. This shift toward an M2‐dominant macrophage profile was sustained through day 7 (Figure S18), indicating that the hydrogel establishes a stable, pro‐regenerative immune microenvironment rather than transiently suppressing inflammation.

The recruitment of endogenous dental pulp stem cells was next evaluated to determine whether immune–redox reprogramming could be translated into effective cellular homing in vivo. Immunofluorescence staining for CD29 revealed a accumulation of CD29‐positive cells at the hydrogel–pulp interface in the DGDL‐Apt group at day 3 (Figure 9D). Quantitative analysis of CD29 fluorescence intensity further confirmed the higher expression in the DGDL‐Apt group (Figure S19C). In contrast, only sparse CD29‐positive signals were detected in the DGDL group without aptamer functionalization, as well as in the LPS and DG control groups. This spatial enrichment of CD29^+^ cells confirms that the conjugated aptamers function as molecular recruitment anchors in vivo, actively concentrating endogenous DPSCs at the injury site during the early reparative window. The early‐stage in vivo findings demonstrate that the DGDL‐Apt hydrogel rapidly reprograms the inflammatory microenvironment and simultaneously initiates targeted endogenous stem cell recruitment. This coordinated early intervention establishes the biological foundation for subsequent high‐quality reparative dentin formation, providing direct temporal and mechanistic linkage between microenvironmental reprogramming and long‐term regenerative outcomes.

## 3 Conclusions

In this study, we present a multifunctional, aptamer‐functionalized porous double‐network hydrogel as an active therapeutic platform for vital pulp therapy. Departing from conventional pulp capping materials that primarily rely on passive physical sealing, this hydrogel is rationally designed to reprogram the inflammatory microenvironment while simultaneously enabling targeted recruitment of endogenous dental pulp stem cells. Mechanistically, the incorporation of dopamine‐derived catechol motifs confers robust redox‐regulatory capacity, effectively restoring oxidative balance and attenuating excessive inflammatory signaling. This redox‐driven immunomodulation promotes a phenotypic shift of macrophages from a pro‐inflammatory M1 state toward a reparative M2 state, thereby establishing a permissive immune niche during the critical early phase of pulp healing. Concurrently, sustained release of bioactive ions from Laponite nanoplates provides pro‐odontogenic biochemical cues that support stem cell differentiation and matrix mineralization. Importantly, conjugation of a high‐affinity CD29‐targeting aptamer enables precise in situ homing of endogenous DPSCs to the injury site, ensuring that the reprogrammed microenvironment is effectively translated into functional tissue regeneration. In a rat model of pulpitis, the DGDL‐Apt hydrogel demonstrated rapid inflammation resolution, efficient endogenous stem cell enrichment and robust formation of a continuous, high‐quality reparative dentin bridge, achieving therapeutic outcomes comparable to the current clinical gold standard. These in vivo results validate that coordinated microenvironmental reprogramming and targeted cellular recruitment are essential for successful pulp preservation. Collectively, this study establishes a new approach for vital pulp therapy by integrating immune–redox regulation with endogenous stem cell homing in a single injectable platform. Beyond dental applications, this strategy highlights a broadly applicable design principle for regenerative biomaterials, in which active microenvironmental control and spatially resolved cell recruitment are leveraged to enable functional tissue preservation and regeneration.

## 4 Experimental Methods

### 4.1 Synthesis of GelMA‐DA

GelMA was synthesized as previously described [14]. Briefly, 0.4 g of lyophilized GelMA was dissolved in 50 mL of MES buffer (0.05 M, pH 5.5) in a 100 mL round‐bottom flask at 50°C. Upon cooling to room temperature, 192 mg of EDC and 116 mg of NHS were added sequentially. The mixture was stirred at room temperature for 30 min under continuous nitrogen purging to activate carboxyl groups and eliminate dissolved oxygen. Separately, 0.4 g of dopamine hydrochloride was dissolved in 2 mL of MES buffer in an amber glass vial, purged with nitrogen for 10 min, and subsequently introduced into the reaction mixture via a syringe. The reaction proceeded in the dark under nitrogen flow for 1 h, followed by continuous stirring at 300 rpm for 24 h in the dark. The resulting solution was transferred to dialysis tubing (MWCO: 8–14 kDa) and dialyzed against acidic deionized water (pH 5–6) for 3 days (with water replaced 4 times daily) to prevent catechol oxidation, followed by dialysis against neutral deionized water for 1 day. Finally, the purified solution was lyophilized to yield GelMA‐DA as a foamy solid, which was stored at −20°C. The successful synthesis and functionalization of GelMA and GelMA‐DA were confirmed by Fourier‐transform infrared spectroscopy (FTIR) and proton nuclear magnetic resonance (^1^H NMR) spectroscopy, respectively.

### 4.2 Preparation of DGDL Macroporous Hydrogel Based on Air‐In‐Water Emulsions

To formulate the precursor solutions, 1% (w/v) salmon testes DNA (M_w_≈1.3 × 10^6^ Da, ≈ 2000 bp; Sigma–Aldrich) and 0.5% (w/v) photoinitiator (I2959) were dissolved in aqueous solutions containing 8% (w/v) of either GelMA or GelMA‐DA under constant stirring at 40°C. Specific fabrication protocols were then employed for each hydrogel group: (1) DG: The DNA/GelMA mixture was subjected to thermal denaturation at 95°C for 5 min, followed by high‐speed homogenization (IKA T10 Basic) to create an emulsion, and subsequently cured via UV photopolymerization (365 nm) for 60 s. (2) DGDA‐N (Non‐porous): The DNA/GelMA‐DA precursor was directly crosslinked via UV irradiation, omitting the thermal and emulsification steps. (3) DGDA: Similar to the DG group, the DNA/GelMA‐DA solution underwent the identical heating (95°C, 5 min) and homogenization procedures prior to UV exposure. (4) DGDL: This group was prepared by dispersing 1% (w/v) Laponite clay nanosheets into the DNA/GelMA‐DA matrix before proceeding with the standard heating, emulsification, and photocrosslinking sequence.

It should be noted that the hydrogel in this study was crosslinked using low‐dose UVA irradiation (365 nm) in combination with a cytocompatible photoinitiator (I2959). Under the optimized conditions (low initiator concentration and short exposure time), previous studies [17, 29] and our experimental results have demonstrated minimal cytotoxicity. In addition, the current system is readily adaptable to visible‐light initiation, which may further improve its clinical translatability.

### 4.3 Structural and Morphological Characterization

The morphology and elemental composition of the DGDL hydrogel were characterized using scanning electron microscopy (SEM; FEI Q25, USA) at an acceleration voltage of 5–10 kV, equipped with energy‐dispersive X‐ray spectroscopy (EDS). Structural details were further examined via transmission electron microscopy (TEM; JEM2100HR, Japan). Surface chemical composition and bonding states were analyzed using X‐ray photoelectron spectroscopy (XPS) using an ESCALAB Xi+ spectrometer (Thermo Fisher Scientific, Waltham, MA, USA), while surface charge properties were evaluated via zeta potential measurements. To visualize the internal pore architecture, GelMA was labeled with FITC, and the emulsified pore structure was imaged using a Zeiss LSM 980 confocal laser scanning microscope (Carl Zeiss, Jena, Germany) to assess porosity and pore size distribution.

### 4.4 Mechanical Characterization

Rheological properties were evaluated using a rheometer (DHR20, TA Instruments) equipped with a 20 mm parallel plate geometry (PP08). Disk‐shaped samples (12 mm in diameter, 8.0 mm in height) were loaded onto the plate. Strain sweeps were conducted (0.01–100% strain, 10 rad/s) to analyze the storage (G') and loss (G'') moduli. Frequency sweeps (0.1–100 rad/s) and time‐dependent rheological behaviors were assessed at a fixed strain of 1%. Additionally, viscosity was measured across shear rates ranging from 0.1 to 100 s^−^^1^.

The self‐healing capability of the DGDL hydrogel was evaluated both macroscopically and quantitatively. For macroscopic observation, heart‐shaped hydrogels were stained with methylene blue or rhodamine, cut in half, and re‐contacted at 37°C for 5 min. Quantitative validation was performed via rheological recovery tests and compressive mechanical assays to determine the restoration of mechanical integrity post‐healing.

The interfacial integration of the DGDL hydrogel within a tooth cavity was examined though SEM. An ex vivo pulp capping model was established using intact human teeth extracted for orthodontic purposes. The study protocol was approved by the Ethics Committee of Guanghua School of Stomatology, Sun Yat‐sen University (Approval No. KQEC‐2025‐057‐01). Standardized Class‐II cavities with simulated pulp chamber exposures were prepared on the teeth using a high‐speed fissure bur under continuous water cooling. Subsequently, the DGDL hydrogel precursor was injected directly into the defect area, ensuring complete coverage of the pulpal floor and axial walls, followed by in situ UV crosslinking to form a solid matrix. To assess the micro‐morphology of the hydrogel‐dentin interface, the samples were lyophilized, coated with a thin layer of gold, and observed using a SEM.

Compressive mechanical properties were assessed using a Instron 5967 universal testing machine (Instron, USA). Cylindrical samples (12 mm in diameter, 8.0 mm in height) were subjected to unconfined compression at a force ramp rate of 0.5 N/min. The compressive modulus was calculated from the slope of the stress–strain curve within the 10–20% strain interval, while toughness was determined by integrating the area under the curve.

The adhesive properties of the DGDL hydrogel to inorganic dental substrates were quantitatively evaluated. Ceramic plates were employed as testing substrates to simulate the rigid, inorganic, hydroxyapatite‐rich environment of human dentin. The DGDL precursor was applied between two overlapping ceramic plates with a standardized hydrogel thickness of 1 mm. To accommodate the specific mechanical configurations, the initial bonded contact area was precisely defined as 20 × 20 mm for the tensile adhesion tests and 20 × 10 mm for the lap‐shear tests. The prepared assemblies were then subjected to in situ UV crosslinking to form a solid adhered joint. Mechanical testing was conducted using a universal testing machine equipped with a 50 N load cell. For the tensile adhesion test, the crosslinked assemblies were pulled apart perpendicularly to the bonded interface at a constant crosshead speed of 10 mm/min until failure. Conversely, for the lap‐shear test, the plates were pulled in opposite directions parallel to the bonded interface at the identical constant crosshead speed. The respective adhesive strengths (tensile and shear) were calculated by dividing the maximum recorded load at failure by the initial bonded area. All mechanical experiments were performed in triplicate to ensure statistical reproducibility.

### 4.5 In Vitro Stability and Ionic Release Profiles

To evaluate in vitro degradation, lyophilized hydrogels of known initial dry weight (W_d_) were immersed in a collagenase solution (1 µg/mL). At scheduled intervals (1, 3, 5, 7, 10, 14, and 21 days), samples were retrieved, lyophilized, and weighed (W_r_). The mass loss ratio was calculated as: (W_d_ – W_r_) / W_d_ × 100%.

Swelling behavior was examined by immersing the equilibrated hydrogels (initial mass M_0_) in PBS at 37°C. At designated time points, swollen samples were weighed (M_t_), and the swelling ratio (Q) was determined using the formula: Q = (M_t_ – M_0_) / M_0_.

To investigate ion release behavior, 600 µL of hydrogel was immersed in 6 mL of PBS within a centrifuge tube and incubated at 37°C under constant shaking. Over a 14‐day period, 500 µL of supernatant was withdrawn at specific time points for analysis and immediately replaced with fresh PBS to maintain a constant volume. The concentrations of released ions (Mg^2+^, Li^+^ and Si^4+^) were quantified using inductively coupled plasma optical emission spectroscopy (ICP‐OES).

### 4.6 Isolation and Culture of Dental Pulp Stem Cells (DPSCs)

DPSCs were isolated from exfoliated teeth obtained from healthy donors who provided informed consent. The study protocol was approved by the Ethics Committee of Guanghua School of Stomatology, Sun Yat‐sen University (Approval No. KQEC‐2025‐057‐01) and conducted in strict accordance with institutional ethical guidelines.

The isolation and culture procedures were performed as previously described [52]. Briefly, dental pulp tissues were extracted from the pulp chamber and minced into small fragments. The tissue fragments were enzymatically digested with a solution containing 3 mg/mL type I collagenase and 3 mg/mL dispase (Sigma–Aldrich, MO, USA) to generate single‐cell suspensions. The resulting cells were seeded into T‐25 culture flasks. DPSCs were cultured in Alpha Minimum Essential Medium (*α‐*MEM; Gibco, Thermo Fisher Scientific, USA) supplemented with 10% fetal bovine serum (FBS; Gibco) and 100 IU/mL penicillin/streptomycin (Sigma–Aldrich). Cultures were maintained in a humidified incubator at 37°C with 5% CO_2_ (Thermo Fisher Scientific). The culture medium was replenished every 3 days to ensure optimal cell growth.

### 4.7 Identification and Characterization of hDPSCs

Single‐cell suspensions were prepared at a density of 10^6^ cells/100 µL in phosphate‐buffered saline (PBS). To prevent nonspecific binding, cells were pre‐incubated with an Fc receptor blocking reagent (BioLegend, San Diego, CA, USA) at 4°C for 10 min. Subsequently, the cells were stained with fluorochrome‐conjugated antibodies against human CD29, CD44, CD45, CD73, CD90, and HLA‐DR (all from BD Biosciences, San Jose, CA, USA) at a 1:100 dilution at 4°C for 30 min in the dark. Following incubation, the cells were washed, resuspended in 500 µL of PBS, and the data was acquired using a BD LSRFortess flow cytometer (BD Biosciences). Data analysis was performed using FlowJo software (Version 10; FlowJo, USA). All experiments were conducted in at least three independent replicates.

Osteogenic differentiation was induced by culturing hDPSCs in *α*‐MEM supplemented with 50 µg/mL ascorbic acid, 10 mM *β*‐glycerophosphate, and 100 nM dexamethasone (all from Sigma–Aldrich, St. Louis, MO, USA). Cells were maintained for 7 or 14 days, with the medium replaced every 3 days. For adipogenic differentiation, hDPSCs were cultured in *α*‐MEM supplemented with 500 µM 3‐isobutyl‐1‐methylxanthine (IBMX), 10 µg/mL insulin, 100 µM indomethacin, and 1 µM dexamethasone. Induction was maintained for 21 days, with medium changes performed every 3 days.

Following the induction periods, cells were fixed with 4% paraformaldehyde (PFA) and subjected to histological staining. Alkaline Phosphatase (ALP) activity was assessed using an ALP staining kit (Sigma–Aldrich) according to the manufacturer's protocol. Calcium nodule formation was visualized using 1% Alizarin Red S (ARS) solution (GL Biochem, Shanghai, China). Adipogenic differentiation was evaluated via Oil Red O staining to detect lipid droplet accumulation. After staining, samples were washed to remove excess dye, and images were captured using an inverted phase‐contrast microscope (Olympus, Tokyo, Japan).

### 4.8 Cytocompatibility and Hemocompatibility Assessment

The proliferation and viability of hDPSCs cultured with hydrogel extracts were evaluated. hDPSCs were seeded into 96‐well plates at a density of 10^3^ cells/well and cultured in conditioned medium containing hydrogel extracts (100 mg/mL).

Cell Proliferation (CCK‐8): Cell proliferation was quantified at days 1, 3, 5, and 7 using the Cell Counting Kit‐8 (CCK‐8) assay. Briefly, CCK‐8 working solution was added to each well, and the cells were incubated for 1 h at 37°C. The optical density (OD) was measured at 450 nm using a microplate reader.

Cell Viability (Live/Dead Assay): Cell viability was visualized after 1, 3, and 5 days of culture using a Live/Dead staining kit. Cells were stained with calcein‐AM (excitation: 488 nm; live cells, green fluorescence) and propidium iodide (PI) (excitation: 561 nm; dead cells, red fluorescence). Representative images were acquired using an inverted fluorescence microscope.

Hemolysis Assay: Sheep whole blood (Hongquanbio, China) was centrifuged at 1,000 rpm for 10 min to isolate erythrocytes. The erythrocytes were washed three times with physiological saline (0.9% NaCl) until the supernatant became clear, and subsequently resuspended in saline to obtain a 2% (v/v) red blood cell (RBC) suspension. For the assay, the RBC suspension was incubated with the various hydrogel samples at 37°C for 2 h. Deionized water (ddH_2_O) and saline served as the positive and negative controls, respectively. Following incubation, the mixtures were centrifuged at 3,000 rpm for 10 min. The optical density (OD) of the supernatant was measured at 545 nm using a microplate reader. The hemolysis ratio (HR) was calculated using the following equation: HR (%) = (OD_sample_ – OD_neg_) / (OD_pos_ – OD_neg_) × 100%, where OD_sample_, OD_pos_ and OD_neg_ represent the absorbance values of the experimental group, positive control, and negative control, respectively.

### 4.9 In Vitro Cellular Response Evaluations

hDPSCs and RAW 264.7 murine macrophages were routinely cultured in complete *α‐*MEM supplemented with 10% FBS and 1% penicillin‐streptomycin at 37°C in a humidified 5% CO_2_ atmosphere. To evaluate the anti‐inflammatory properties of the hydrogels, an in vitro inflammatory model was established using both hDPSCs and RAW 264.7 cells. Briefly, cells were seeded into 6‐well plates at a density of 2 × 10^5^ cells/well and allowed to attach overnight. To induce inflammation, the cells were stimulated with 1 µg/mL lipopolysaccharide (LPS) for 3 h, while the blank control group (Control) received standard medium without LPS. Following the 3‐h stimulation, all wells were gently washed 3 times with PBS. The Control and LPS groups were then cultured in fresh standard medium, while the experimental groups were cultured in media supplemented with the respective hydrogel extracts. The cells were further incubated for 6 or 12 h before harvesting for downstream analyses.

To assess the odontogenic potential, hDPSCs were seeded in 6‐well plates at a density of 1 × 10^5^ cells/mL. Upon reaching approximately 80% confluence, the standard culture medium was replaced with specific odontogenic induction media. The cells were divided into two primary groups: the Control group was cultured in a standard induction medium based on *α*‐MEM, and the hydrogel experimental groups, cultured in customized induction media formulated using the respective hydrogel extracts. All induction media were supplemented with a standardized odontogenic supplements comprising 50 µg/mL ascorbic acid, 10 mM *β*‐glycerophosphate, and 100 nM dexamethasone. The induction media were refreshed every 3 days. For downstream mRNA and protein expression analyses, the cultured cells were harvested at day 5 post‐induction. For the morphological evaluation of mineralized matrix formation, parallel samples were maintained and subjected to specific staining assays at day 7 and day 14.

#### 4.9.1 Real‐Time Quantitative Polymerase Chain Reaction (qRT‐PCR)

Total RNA was extracted using RNAzol reagent (MRC, Cincinnati, OH, USA) according to the manufacturer's protocol. RNA concentration and purity were assessed via spectrophotometry, and samples with an A_260_/A_280_ ratio between 1.8 and 2.0 were used. cDNA synthesis was performed using the ABScript III RT Master Mix (ABclonal, Wuhan, China). Quantitative real‐time PCR (qPCR) was conducted on a LightCycler 480 system (Roche, Basel, Switzerland) with the following thermal cycling conditions: initial denaturation at 95°C for 5 min, followed by 40 cycles of denaturation at 95°C for 10 s and annealing/extension at 60°C for 30 s. All reactions were performed in triplicate. The specific primer sequences are listed in Table 4. Relative gene expression levels were calculated using the 2^−ΔΔCt^ method, normalized to GAPDH.

**TABLE 4:** Primer sequences for qRT‐PCR.

Gene	Forward Primer (5’‐3’)	Reverse Primer (3’‐5’)
H‐GAPDH	GGAGCGAGATCCCTCCAAAAT	GGCTGTTGTCATACTTCTCATGG
H‐DSPP	TTTGGGCAGTAGCATGGGC	CCATCTTGGGTATTCTCTTGCCT
H‐DMP1	CTCCGAGTTGGACGATGAGG	TCATGCCTGCACTGTTCATTC
H‐BMP2	ACCCGCTGTCTTCTAGCGT	TTTCAGGCCGAACATGCTGAG
H‐OCN	CACTCCTCGCCCTATTGGC	CCCTCCTGCTTGGACACAAAG
H‐IL‐6	ACTCACCTCTTCAGAACGAATTG	CCATCTTTGGAAGGTTCAGGTTG
H‐IL‐8	TTTTGCCAAGGAGTGCTAAAGA	AACCCTCTGCACCCAGTTTTC
H‐TNF‐*α*	CCTCTCTCTAATCAGCCCTCTG	GAGGACCTGGGAGTAGATGAG
H‐IL‐1*β*	ATGATGGCTTATTACAGTGGCAA	GTCGGAGATTCGTAGCTGGA
H‐NANOG	TTTGTGGGCCTGAAGAAAACT	AGGGCTGTCCTGAATAAGCAG
H‐SOX	CATCACCCACAGCAAATGAC	TTTTTCGTCGCTTGGAGACT
H‐KLF4	CCCACATGAAGCGACTTCCC	CAGGTCCAGGAGATCGTTGAA
H‐Pou5f1	CTTGAATCCCGAATGGAAAGGG	GTGTATATCCCAGGGTGATCCTC
M‐GAPDH	GCAAAGTGGAGATTGTTGCC	TGGAAGATGGTGATGGGCTT
M‐IL‐6	CTGCAAGAGACTTCCATCCAG	AGTGGTATAGACAGGTCTGTTGG
M‐TNF‐*α*	CCACCACGCTCTTCTGTCTA	GGTCTGGGCCATAGAACTGA
M‐Arg1	GCTGGGAAGGAAGAAAAAGGC	TGCCGTGTTCACAGTACTCT
M‐Fizz	CAGAAGGCACAGCAGTCTTG	GGGTATTAGCTCCTGTCCCC

#### 4.9.2 Western Blot Analysis

Total protein was extracted using RIPA lysis buffer (Beyotime, China) supplemented with protease and phosphatase inhibitors (Cwbiotec, China) on ice for 30 min. Protein concentration was quantified using a BCA assay kit (ComWin Biotech, China). Equal amounts of protein (25 µg) were separated via 10% SDS‐PAGE and transferred onto polyvinylidene fluoride (PVDF) membranes (Millipore, USA). The membranes were blocked with 5% bovine serum albumin (BSA) for 1 h at room temperature and subsequently incubated overnight at 4°C with primary antibodies against DSPP (1:1000; Affinity, USA), DMP1 (1:1000; ABclonal, China), and GAPDH (1:50,000; ABclonal). Following washing, the membranes were incubated with HRP‐conjugated secondary antibodies. Protein bands were visualized using an enhanced chemiluminescence (ECL) kit (Millipore) and imaged using a chemiluminescence detection system. GAPDH served as the internal control.

#### 4.9.3 ELISA Analysis of Cytokine Levels

The concentrations of human (IL‐1*β*, TNF‐*α*) and mouse (IL‐6, TNF‐*α*) cytokines were quantified using specific ELISA kits (R&D Systems, USA) in strict accordance with the manufacturer's instructions. Briefly, standards and samples were added to antibody‐precoated 96‐well plates and incubated at 37°C for 2 h. Following washing, biotinylated detection antibodies were added (1 h at 37°C), followed by incubation with streptavidin‐HRP conjugate (30 min at 37°C). The enzymatic reaction was visualized using TMB substrate for 15–30 min in the dark and terminated by the addition of a stop solution. Absorbance was measured at 450 nm using a microplate reader. Cytokine concentrations were calculated based on standard curves, with all samples analyzed in triplicate. Final results were normalized to total protein content as determined by the BCA assay.

#### 4.9.4 Measurement of Nitric Oxide (NO) Production

The concentration of nitric oxide (NO) in the cell culture supernatants was determined by a Griess Reagent Kit (S0021; Beyotime, Shanghai, China) according to the manufacturer's instructions. Briefly, the cell culture supernatant was collected and centrifuged at 1000 × g for 5 min to remove cell debris. 50 µL of the supernatant was mixed with an equal volume of Griess Reagent I and Griess Reagent II in a 96‐well plate at room temperature. Absorbance was then measured at 540 nm using a microplate reader, and NO concentrations were calculated based on a sodium nitrite standard curve.

### 4.10 Evaluation of Antioxidant Activity

DPPH Free Radical Scavenging Assay: Briefly, 200 µL of the hydrogel was mixed with 200 µL of a 0.2 mM DPPH ethanolic solution in a 96‐well microplate. The mixture was incubated in the dark at 37°C for 30 min. Absorbance was measured at 517 nm using a microplate reader. The DPPH scavenging activity was calculated using the following equation: Scavenging Activity (%) = [1 – (A_sample_ – A_background_)/(A_control_ – A_background_)] × 100%, where A_sample_, A_control_ and A_background_ represent the absorbance of the sample, the control (DPPH + solvent), and the background (sample + solvent), respectively.

ABTS Radical Scavenging Assay: The ABTS^●+^ working solution was generated by reacting 7 mM ABTS with 2.45 mM potassium persulfate and incubating the mixture in the dark at room temperature for 12 h. Prior to use, the solution was diluted with ethanol to achieve an absorbance of 0.70 ± 0.02 at 734 nm. Subsequently, 20 µL of the hydrogel was mixed with 180 µL of the diluted ABTS^●+^ solution in a 96‐well microplate and incubated for 5 min in the dark. Absorbance was recorded at 734 nm. The scavenging activity was determined as follows: Scavenging Activity (%) = (A_control_ – A_sample_) / A_control_ × 100%.

H_2_O_2_ Scavenging Assay: Briefly, 500 µL of 10 mM H_2_O_2_ solution was mixed with 100 µL of the various hydrogels, while deionized water served as the control. After incubation at 37°C for 12 h, 100 µL of 40 mM DCFH solution was added to each sample, followed by a further 2 h incubation in the dark. The reaction mixture (100 µL) was then transferred to a 96‐well plate, and fluorescence intensity was measured at an emission wavelength of 525 nm. The H_2_O_2_ residual rate was calculated using the formula: Residual Rate (%) = A_sample_/A_control_ × 100%. All antioxidant assays were performed in triplicate.

### 4.11 Measurement of Intracellular ROS Levels

Intracellular reactive oxygen species (ROS) generation was assessed using a commercial ROS assay kit (Phygene, China). Briefly, DPSCs or RAW264.7 macrophages were seeded 24 h before stimulated with 1 µg/mL LPS for 24 h to induce oxidative stress, followed by treatment with hydrogel extracts for an additional 6 h. Subsequently, cells were incubated with 10 µM 2′,7′‐dichlorodihydrofluorescein diacetate (DCFH‐DA) at 37°C for 30 min in the dark. After washing with PBS to remove extracellular dye, intracellular fluorescence was imaged using a confocal laser scanning microscope (CLSM) (Excitation: 488 nm; Emission: 525 nm). The fluorescence intensity was quantified to determine relative ROS levels.

### 4.12 Selection and Computational Prediction of Aptamer Structures

Based on prior literature, three DNA aptamer candidates demonstrating high affinity and specificity toward stem cells or the surface markers CD29 and CD44 were selected. The sequences (5'–3') are detailed in Table 5.

**TABLE 5:** Sequences of Selected Aptamer Candidates.

Aptamer Candidate	Sequence (5’ to 3’)
Aptamer 19S	AGGTCAGATGAGGAGGGGGACTTAGGACTGGGTTTATGACCTATGCGTG
Aptamer CD29	ATCCAGAGTGACGCAGCATGGGGGTAGTGGTGGTTAGGAGTGGAGGCGAGGAGAGCGGTGGACACGGTGGCTTAGT
Aptamer CD44	CCAAGGCCTGCAAGGGAACCAAGGACACAGTTTTTTTTTT

The secondary and tertiary structures of the selected aptamers were predicted using computational modeling tools. First, the secondary structures were predicted using the mfold web server (http://unafold.rna.albany.edu/?q = mfold). The folding temperature was set to 37°C, and ionic conditions were adjusted to 1 M Na^+^ and 0 M Mg^2+^ to simulate physiological conditions. The structure exhibiting the minimum free energy was selected for further analysis. Subsequently, the secondary structure data was utilized to generate 3D tertiary structural models via the RNA Composer automated server (http://rnacomposer.cs.put.poznan.pl/). Finally, the resulting PDB files were imported into the PyMOL Molecular Graphics System (Schrödinger, LLC) for structural visualization and rendering.

### 4.13 Characterization of Aptamer Binding Performance

Flow Cytometric Analysis of Specificity and Affinity: To evaluate binding specificity and affinity, flow cytometry was performed. Washing buffer was prepared by supplementing 1 L of DPBS with 4.5 g glucose and 5 mM MgCl. Binding buffer consisted of washing buffer further supplemented with 0.1 mg/mL tRNA and 1 mg/mL BSA to minimize nonspecific binding. DPSCs (1 × 10^5^ cells) were washed and incubated with FAM‐labeled aptamers in 200 µL of binding buffer at 37°C for 60 min. For specificity analysis, a fixed concentration of 250 nM was used. For equilibrium dissociation constant (K_d_) determination, cells were incubated with a concentration gradient of aptamers. Following incubation, cells were washed twice with 1 mL of washing buffer and resuspended in 500 µL of PBS. The samples were analyzed using a BD LSRFortessa flow cytometer (BD Biosciences, Franklin Lakes, NJ, USA). Data analysis and fluorescence intensity quantification were performed using FlowJo software (Version 10; FlowJo, USA). The K_d_ value was determined by fitting the dependence of mean fluorescence intensity (Y) on aptamer concentration (X) to the non‐linear regression equation: Y = (B_max_ × X) / (K_d_ + X), where B_max_ represents the maximum binding capacity.

Cellular Visualization via CLSM: Human DPSCs and Rat DPSCs were seeded in 20‐mm glass‐bottom dishes (1 × 10^5^ cells/dish) and cultured for 24 h. Cells were washed with 4°C washing buffer and incubated with FAM‐labeled aptamers or a random library (250 nM) in 200 µL of binding buffer at 37°C for 3 h. After washing three times with 1 mL of washing buffer, cellular fluorescence was imaged using a Zeiss LSM 980 confocal microscope.

### 4.14 In Vitro Targeted Recruitment Assay

Cell migration was assessed using Transwell assays (Corning, NY, USA; 8.0 µm pore size). DPSCs were resuspended in serum‐free medium at a density of 1 × 10^5^ cells/mL and treated with 100 nM of the selected aptamers. Untreated cells and cells treated with random library aptamers were served as negative controls. A 200 µL of the cell suspension was seeded into the upper chamber, while 600 µL of medium supplemented with 5% FBS was added to the lower chamber to serve as a chemoattractant. In parallel experiments designed to evaluate the chemoattractive potential of the materials, 200 µL of the respective hydrogel formulations were placed in the lower chamber, and the identical experimental protocol was followed. Following a 24 h incubation at 37°C, non‐migrated cells on the upper surface of the membrane were removed using a cotton swab. The migrated cells on the lower surface were fixed with 4% paraformaldehyde and stained with 0.1% crystal violet. Stained cells were imaged using an optical microscope, and migration was quantified by counting cells in five randomly selected fields per well using Image J software.

To evaluate the specific capture efficiency and selectivity of the aptamer‐functionalized hydrogels, an in vitro cell capture assay was performed. Non‐emulsified (non‐porous) GelMA hydrogels were utilized for this assay to ensure a uniform focal plane for accurate morphological observation and cell quantification. The hydrogel precursors, formulated either without aptamer (Control) or conjugated with Apt‐CD29, Apt‐CD44, or Apt‐19S, were crosslinked at the bottom of confocal cell culture dishes. hDPSCs were uniformly seeded onto the respective hydrogel surfaces at a density of 1 × 10^4^ cells/well. The cells were incubated at 37°C for 6 h. This specific 6‐h time point was strategically selected to evaluate the rapid, specific binding mediated by the aptamer‐receptor interactions while minimizing the background interference of non‐specific, integrin‐mediated cell adhesion intrinsically driven by the RGD sequences within the GelMA matrix. Following incubation, the hydrogel surfaces were gently washed with PBS to remove any non‐adherent cells. The remaining captured cells were observed using confocal laser scanning microscope, and the number of adherent cells per field of view was quantified using Image J software.

To visually and qualitatively evaluate the capability of the hydrogels to actively recruit surrounding cells, an in vitro macroscopic recruitment assay was established. Briefly, defined regions of either DGDL or DGDL‐Apt hydrogel precursors were patterned in the central area of 6‐well culture plates. The patterned precursors were immediately stabilized via in situ UV crosslinking (365 nm, 1 min). Subsequently, hDPSCs were seeded uniformly into the surrounding regions of the wells at a density of 5 × 10^4^ cells/well. The cells were cultured in standard complete medium at 37°C with 5% CO_2_. Cell migration from the periphery toward the central hydrogel zone was continuously monitored. Representative images of the hydrogel‐cell boundaries were captured at 12 h and 24 h post‐seeding using an inverted phase‐contrast microscope.

### 4.15 Rat Model of Pulpitis and Direct Pulp Capping

Seven‐week‐old male Sprague‐Dawley (SD) rats were obtained from the Animal Experiment Center of Sun Yat‐sen University. All animal procedures were conducted in strict accordance with institutional guidelines and approved by the Institution Animal Care and Use Committee (IACUC) of Sun Yat‐sen University (Approval No. SYSU‐IACUC‐2024‐002294).

Rats were anesthetized via intraperitoneal injection of sodium pentobarbital (40 mg/kg). Following disinfection with 1% sodium hypochlorite (NaClO), a cavity was prepared on the occlusal surface of the maxillary first molar using a 1/4 round bur under water cooling. Pulp exposure was mechanically created and sequentially enlarged using #10, #20, #30, and #40 stainless steel K‐files under 2.5× magnification. The cavity was subsequently rinsed with 1% NaClO and sterile PBS to achieve hemostasis. To induce pulpitis, 2 µL of lipopolysaccharide (LPS) (10 mg/mL; InvivoGen, France) was applied directly to the exposed pulp. The rats were randomly assigned to the following groups (n = 6 per group per time point): (1) Negative Control: Intact teeth without pulp exposure; (2) Inflammation Group: Exposed pulp treated with LPS and capped with a sterile gelatin sponge; (3) Hydrogel Treatment Groups: LPS‐stimulated pulp capped with 2 µL of DG, DGDL, or DGDL‐Apt hydrogels, followed by in situ photocrosslinking using a 365 nm UV lamp for 1 min; (4) Positive Control (iRoot BP): LPS‐stimulated pulp capped with iRoot BP Plus (Innovative BioCeramix, Canada). All cavities were sealed with a light‐cured composite resin (3M ESPE, St. Paul, MN, USA). Maxillae were harvested at 3 days, 7 days, 4 weeks, and 8 weeks post‐operation and fixed in 4% paraformaldehyde (PFA) for 24 h for histological analysis.

### 4.16 Micro‐Computed Tomography (Micro‐CT) Evaluation

Samples harvested at 4 and 8 weeks post‐treatment were scanned using a micro‐CT system (Scanco Medical, Brüttisellen, Switzerland). The scanning parameters were set at a voltage of 70 kVp, a current of 114 µA, and a power of 8 W, with an effective pixel size of 10 µm. A semi‐quantitative grading system was employed to assess the dentin bridge based on three criteria: continuity, morphology, and thickness [73]. Each parameter was graded to generate a comprehensive histological score. All sample results were evaluated by two independent calibrated examiners who were blinded to the group assignment.

### 4.17 Histological Processing and Staining

Harvested maxillae were fixed in 4% PFA for 24 h and rinsed thoroughly with running water. Decalcification was performed in 10% EDTA (pH 7.4) at room temperature for approximately 8 weeks, with the solution refreshed every 3 days. Following decalcification, samples were dehydrated through a graded ethanol series, cleared in xylene, and embedded in paraffin. Serial sections (4 µm thick) were prepared for histological analysis.

Hematoxylin and Eosin (H&E) Staining: H&E staining was performed to evaluate general tissue morphology and inflammatory infiltration. Sections were deparaffinized, rehydrated, and stained with hematoxylin for 5 min to visualize nuclei. After rinsing in running tap water, sections were counterstained with eosin for 2 min. Subsequently, the slides were dehydrated through an ascending ethanol series (70%, 80%, 90%, 100%), cleared in xylene, and mounted with neutral balsam.

Masson's Trichrome Staining: To evaluate collagen deposition and matrix formation, sections were subjected to Masson's Trichrome staining according to the manufacturer's protocol (Solarbio, China). Briefly, nuclei were stained with Weigert's iron hematoxylin for 5 min. Sections were then differentiated with an acid alcohol solution, stained with Biebrich scarlet‐acid fuchsin solution, and treated with phosphomolybdic‐phosphotungstic acid. Finally, collagen fibers were stained with aniline blue for 5 min.

Immunofluorescence (IF) Staining: Paraffin‐embedded sections were deparaffinized, rehydrated, and subjected to antigen retrieval via microwave heating in citrate buffer (pH 6.0). To minimize nonspecific binding, sections were blocked with 3% BSA for 30 min at room temperature. Subsequently, the sections were incubated overnight at 4°C with primary antibodies targeting iNOS (1:1000; Proteintech), Arg‐1 (1:500; Proteintech) and CD29 (1:100; Huabio). Following PBS washing, fluorophore‐conjugated secondary antibodies were applied according to the manufacturer's protocol. Nuclei were counterstained with DAPI for 5 min.

Immunohistochemistry (IHC) Staining: For IHC analysis, sections underwent deparaffinization and citrate buffer‐mediated antigen retrieval as described above. Endogenous peroxidase activity was quenched by incubation with 3% H_2_O_2_ for 10 min. After blocking with 3% BSA (30 min, RT), sections were probed with primary antibodies against IL‐6 (1:200; Affinity Biosciences) and CD206 (1:5000; Proteintech) overnight at 4°C. The sections were then incubated with HRP‐conjugated secondary antibodies. Immunoreactivity was visualized using DAB substrate. Sections were counterstained with hematoxylin, dehydrated through a graded ethanol series, cleared in xylene, and mounted with neutral balsam.

All stained slides were digitally scanned using a high‐resolution pathology scanner (Aperio AT2; Leica, Vista, CA, USA).

### 4.18 Statistical Analysis

Statistical analyses were performed using GraphPad Prism 10.3 (GraphPad Software, La Jolla, CA, USA). All quantitative data are presented as the mean ± standard deviation (SD). Differences between groups were evaluated using one‐way ANOVA followed by Tukey's multiple comparisons test. For micro‐CT evaluations for dentinal bridge continuity, morphology and thickness, scoring data are expressed. Differences in histological grades between groups were analyzed using the non‐parametric Kruskal‐Wallis test, followed by Dunn's multiple comparisons test. A *p*‐value of < 0.05 was considered statistically significant (*p < 0.05, **p < 0.01, ***p < 0.001). All experiments were performed in at least three independent replicates.

## Conflicts of Interest

The authors declare no conflicts of interest.

## Supporting information




**Supporting File**: advs75497‐sup‐0001‐SuppMat.docx.

## Data Availability

The data that supports our findings in this study remains available from the corresponding author upon reasonable request.

## References

[1] I. A. Mejàre , S. Axelsson , T. Davidson , et al., “Diagnosis of the Condition of the Dental Pulp: A Systematic Review,” International Endodontic Journal 45 (2012): 597–613. 22329525 10.1111/j.1365-2591.2012.02016.x

[2] Z. Xie , Z. Shen , P. Zhan , et al., “Functional Dental Pulp Regeneration: Basic Research and Clinical Translation,” International Journal of Molecular Sciences 22 (2021): 8991. 34445703 10.3390/ijms22168991 PMC8396610

[3] J. J. Segura‐Egea , D. Cabanillas‐Balsera , J. Martín‐González , and L. T. A. Cintra , “Impact of Systemic Health on Treatment Outcomes in Endodontics,” International Endodontic Journal 56, no. 2 (2023): 219–235. 35752972 10.1111/iej.13789

[4] L. M. Lin , D. Ricucci , T. M. Saoud , A. Sigurdsson , and B. Kahler , “Vital Pulp Therapy of Mature Permanent Teeth With Irreversible Pulpitis From the Perspective of Pulp Biology,” Australian Endodontic Journal 46 (2020): 154–166. 31865629 10.1111/aej.12392

[5] M. Skitioui , A. Seck , S. O. Niang , A. Fikhar , and B. Touré , “The Treatment of Mature Permanent Teeth With Irreversible Pulpitis by Cervical Pulpotomy: A Systematic Review,” Australian Endodontic Journal 49, no. 1 (2023): 488–493. 36149016 10.1111/aej.12694

[6] H. F. Duncan , “Present Status and Future Directions‐Vital Pulp Treatment and Pulp Preservation Strategies,” International Endodontic Journal 55, no. 3 (2022): 497–511. 35080024 10.1111/iej.13688 PMC9306596

[7] D. Ricucci , J. F. Siqueira Jr. , Y. Li , and F. R. Tay , “Vital Pulp Therapy: Histopathology and Histobacteriology‐Based Guidelines to Treat Teeth With Deep Caries and Pulp Exposure,” Journal of Dentistry 86 (2019): 41–52. 31121241 10.1016/j.jdent.2019.05.022

[8] X. Wang , Y. Xiao , W. Song , et al., “Clinical Application of Calcium Silicate‐Based Bioceramics in Endodontics,” Journal of translational medicine 21 (2023): 853. 38007432 10.1186/s12967-023-04550-4 PMC10676601

[9] L. Qiao , X. Zheng , C. Xie , et al., “Bioactive Materials in Vital Pulp Therapy: Promoting Dental Pulp Repair Through Inflammation Modulation,” Biomolecules 15 (2025): 258. 40001561 10.3390/biom15020258 PMC11853510

[10] M. Chung , S. Lee , D. Chen , et al., “Effects of Different Calcium Silicate Cements on the Inflammatory Response and Odontogenic Differentiation of Lipopolysaccharide‐Stimulated Human Dental Pulp Stem Cells,” Materials 12 (2019): 1259. 30999582 10.3390/ma12081259 PMC6514726

[11] M. C. Wang , K. W. Chang , S. C. Lin , and P. S. Hung , “Biodentine but not MTA Induce DSPP Expression of Dental Pulp Cells With Different Severity of LPS‐Induced Inflammation,” Clinical Oral Investigations 27 (2023): 1207–1214. 36208328 10.1007/s00784-022-04734-0

[12] T. Thambi , Y. Li , and D. S. Lee , “Injectable Hydrogels for Sustained Release of Therapeutic Agents,” Journal of Controlled Release 267 (2017): 57–66. 28827094 10.1016/j.jconrel.2017.08.006

[13] Z. Wang , D. Ma , J. Liu , et al., “4D Printing Polymeric Biomaterials for Adaptive Tissue Regeneration,” Bioactive materials 48 (2025): 370–399. 40083775 10.1016/j.bioactmat.2025.01.033 PMC11904411

[14] K. Yue , G. Trujillo‐de Santiago , M. M. Alvarez , et al., “Synthesis, Properties, and Biomedical Applications of Gelatin Methacryloyl (GelMA) Hydrogels,” Biomaterials 73 (2015): 254–271. 26414409 10.1016/j.biomaterials.2015.08.045 PMC4610009

[15] Y. Shao , H. Jia , T. Cao , and D. Liu , “Supramolecular Hydrogels Based on DNA Self‐Assembly,” Accounts of Chemical Research 50 (2017): 659–668. 28299927 10.1021/acs.accounts.6b00524

[16] Q. Yang , Y. Miao , J. Luo , Y. Chen , and Y. Wang , “Amyloid Fibril and Clay Nanosheet Dual‐Nanoengineered DNA Dynamic Hydrogel for Vascularized Bone Regeneration,” ACS Nano 17 (2023): 17131–17147. 37585498 10.1021/acsnano.3c04816

[17] M. Xie , Y. Chen , Q. Yang , et al., “Nano‐Enabled DNA Supramolecular Sealant for Soft Tissue Surgical Applications,” Nano Today 50 (2023): 101825.

[18] Y. Miao , Y. Chen , J. Luo , et al., “Black Phosphorus Nanosheets‐Enabled DNA Hydrogel Integrating 3D‐Printed Scaffold for Promoting Vascularized Bone Regeneration,” Bioactive Materials 21 (2023): 97–109. 36093326 10.1016/j.bioactmat.2022.08.005 PMC9417961

[19] X. Wu , F. Wang , R. Li , et al., “Enzyme‐Programmable DNA‐PEG Hydrogel Spatiotemporally Regulates Bone Regeneration Microenvironment,” Advanced Materials 38 (2026): 14461. 10.1002/adma.202514461 41178145

[20] M. Zhu , H. Zhang , Q. Zhou , et al., “Dynamic GelMA/DNA Dual‐Network Hydrogels Promote Woven Bone Organoid Formation and Enhance Bone Regeneration,” Advanced Materials 37 (2025): 2501254. 10.1002/adma.202501254 40123197

[21] Y. Miao , X. Liu , J. Luo , et al., “Double‐Network DNA Macroporous Hydrogel Enables Aptamer‐Directed Cell Recruitment to Accelerate Bone Healing,” Advanced Science 11 (2024): 2303637. 37949678 10.1002/advs.202303637 PMC10767401

[22] Y. Chen , M. Chai , C. Xuan , et al., “Tuning the Properties of Surgical Polymeric Materials for Improved Soft‐Tissue Wound Closure and Healing,” Progress in Materials Science 143 (2024): 101249.

[23] Q. Yang , Y. Miao , J. Luo , et al., “Nanofibril‐Structured Granular Hydrogels Harness Stem Cell Retention and Immunoregulation in Diabetic Microenvironment,” ACS Nano 19 (2025): 6795–6814. 39932571 10.1021/acsnano.4c11414

[24] M. Zhu , H. Zhang , Q. Zhou , et al., “Dynamic GelMA/DNA Dual‐Network Hydrogels Promote Woven Bone Organoid Formation and Enhance Bone Regeneration,” Advanced Materials 37 (2025): 2501254. 10.1002/adma.202501254 40123197

[25] V. Karageorgiou and D. Kaplan , “Porosity of 3D Biomaterial Scaffolds and Osteogenesis,” Biomaterials 26 (2005): 5474–5491. 15860204 10.1016/j.biomaterials.2005.02.002

[26] P. Zhang , J. Yang , Z. Wang , et al., “An Injectable Self‐Lubricating Supramolecular Polymer Hydrogel Loaded With Platelet Lysate to Boost Osteoarthritis Treatment,” Journal of Controlled Release 376 (2024): 20–36. 39362609 10.1016/j.jconrel.2024.09.052

[27] D. W. Hutmacher , “Scaffolds in Tissue Engineering Bone and Cartilage,” Biomaterials 21 (2000): 2529–2543. 11071603 10.1016/s0142-9612(00)00121-6

[28] J. R. Jones and L. L. Hench , “Factors Affecting the Structure and Properties of Bioactive Foam Scaffolds for Tissue Engineering,” Journal of Biomedical Materials Research Part B: Applied Biomaterials 68 (2004): 36–44. 14689494 10.1002/jbm.b.10071

[29] M. Xie , Z. Zheng , S. Pu , et al., “Macroporous Adhesive Nano‐Enabled Hydrogels Generated from Air‐in‐Water Emulsions,” Macromolecular Bioscience 22 (2022): 2100491. 10.1002/mabi.202100491 35080348

[30] J. W. Kim , S. H. Han , Y. H. Choi , et al., “Recent Advances in the Microfluidic Production of Functional Microcapsules by Multiple‐Emulsion Templating,” Lab on A Chip 22 (2022): 2259–2291. 35608122 10.1039/d2lc00196a

[31] B. P. Binks and R. Murakami , “Phase Inversion of Particle‐Stabilized Materials From Foams to Dry Water,” Nature Materials 5 (2006): 865–869. 17041582 10.1038/nmat1757

[32] M. Xu , Q. Li , M. Xie , et al., “Engineering Air‐In‐Water Emulsion as Adaptable Multifunctional Sealant,” Chemical Engineering Journal 429 (2022): 132200.

[33] M. A. Mudassir , H. Z. Aslam , T. M. Ansari , H. Zhang , and I. Hussain , “Fundamentals and Design‐Led Synthesis of Emulsion‐Templated Porous Materials for Environmental Applications,” Advanced Science 8 (2021): 2102540. 34553500 10.1002/advs.202102540 PMC8596121

[34] S. Liu , M. Jin , Y. Chen , et al., “Air‐In‐Water Emulsion Solely Stabilized by Gelatin Methacryloyl and Templating for Macroporous Nanocomposite Hydrogels,” Macromolecular Chemistry and Physics 220 (2019): 1800500.

[35] M. Xu , Q. Li , Z. Fang , et al., “Conductive and Antimicrobial Macroporous Nanocomposite Hydrogels Generated From Air‐In‐Water Pickering Emulsions for Neural Stem Cell Differentiation and Skin Wound Healing,” Biomaterials Science 8 (2020): 6957–6968. 33103177 10.1039/d0bm01466d

[36] Y. Ma , X. Wang , T. Su , et al., “Recent Advances in Macroporous Hydrogels for Cell Behavior and Tissue Engineering,” Gels 8 (2022): 606. 36286107 10.3390/gels8100606 PMC9601978

[37] J. Michálek , M. Prádný , A. Artyukhov , M. Slouf , and K. Smetana Jr. , “Macroporous Hydrogels Based on 2‐Hydroxyethyl Methacrylate. Part III. Hydrogels as Carriers for Immobilization of Proteins,” Journal of Materials Science: Materials in Medicine 16 (2005): 783–786. 15965750 10.1007/s10856-005-2617-2

[38] X. Shi , X. Hu , N. Jiang , and J. Mao , “Regenerative Endodontic Therapy: From Laboratory Bench to Clinical Practice,” Journal of Advanced Research 72 (2025): 229–263. 38969092 10.1016/j.jare.2024.07.001 PMC12147620

[39] L. Pang , H. Jin , Z. Lu , et al., “Treatment With Mesenchymal Stem Cell‐Derived Nanovesicle‐Containing Gelatin Methacryloyl Hydrogels Alleviates Osteoarthritis by Modulating Chondrogenesis and Macrophage Polarization,” Advanced Healthcare Materials 12 (2023): 2300315. 10.1002/adhm.202300315 36848378

[40] J. L. Liesveld , N. Sharma , and O. S. Aljitawi , “Stem Cell Homing: From Physiology to Therapeutics,” Stem Cells 38 (2020): 1241–1253. 32526037 10.1002/stem.3242

[41] Y. Xiao , T. Pan , W. Da , et al., “Aptamer‐Drug Conjugates‐Loaded Bacteria for Pancreatic Cancer Synergistic Therapy,” Signal Transduction and Targeted Therapy 9 (2024): 272. 39397032 10.1038/s41392-024-01973-3 PMC11471780

[42] S. Yang , J. Wen , H. Li , et al., “Aptamer‐Engineered Natural Killer Cells for Cell‐Specific Adaptive Immunotherapy,” Small 15 (2019): 1900903. 10.1002/smll.201900903 PMC6541510 31026116

[43] X. Wang , B. Jia , K. Lee , et al., “Biomimetic Bacterial Capsule for Enhanced Aptamer Display and Cell Recognition,” Journal of the American Chemical Society 146 (2024): 868–877. 38153404 10.1021/jacs.3c11208

[44] C. E. Brunchi and S. Morariu , “Laponite(®)‐From Dispersion to Gel‐Structure, Properties, and Applications,” Molecules 29 (2024): 2823. 38930887 10.3390/molecules29122823 PMC11206873

[45] J. W. Davern , L. Hipwood , L. J. Bray , C. Meinert , and T. J. Klein , “Addition of Laponite to Gelatin Methacryloyl Bioinks Improves the Rheological Properties and Printability to Create Mechanically Tailorable Cell Culture Matrices,” APL Bioengineering 8 (2024): 016101. 38204454 10.1063/5.0166206 PMC10776181

[46] J. K. Carrow , L. M. Cross , R. W. Reese , et al., “Widespread Changes in Transcriptome Profile of Human Mesenchymal Stem Cells Induced by Two‐Dimensional Nanosilicates,” PNAS 115 (2018): e3905–e3913. 29643075 10.1073/pnas.1716164115 PMC5924886

[47] W. Li , Z. Su , Y. Hu , et al., “Mussel‐Inspired Methacrylated Gelatin‐Dopamine/Quaternized Chitosan/Glycerin Sponges With Self‐Adhesion, Antibacterial Activity, and Hemostatic Ability for Wound Dressings,” International Journal of Biological Macromolecules 241 (2023): 124102. 36958445 10.1016/j.ijbiomac.2023.124102

[48] A. Gonzalez‐Pujana , M. Igartua , R. M. Hernandez , and E. Santos‐Vizcaino , “Laponite Nanoclays for the Sustained Delivery of Therapeutic Proteins,” European Journal of Pharmaceutical Sciences 201 (2024): 106858. 39033884 10.1016/j.ejps.2024.106858

[49] K. Haraguchi and T. Takehisa , “Nanocomposite Hydrogels: A Unique Organic–Inorganic Network Structure with Extraordinary Mechanical, Optical, and Swelling/De‐swelling Properties,” Advanced Materials 14 (2002): 1120–1124.

[50] B. P. Binks , “Particles as Surfactants—Similarities and Differences,” Current Opinion in Colloid & Interface Science 7 (2002): 21–41.

[51] C. Li , Q. Liu , Z. Mei , et al., “Pickering Emulsions Stabilized by Paraffin Wax and Laponite Clay Particles,” Journal of Colloid & Interface Science 336 (2009): 314–321. 19428022 10.1016/j.jcis.2009.03.080

[52] T. N. Hunter , R. J. Pugh , G. V. Franks , and G. J. Jameson , “The Role of Particles in Stabilising Foams and Emulsions,” Advances in Colloid and Interface Science 137 (2008): 57–81. 17904510 10.1016/j.cis.2007.07.007

[53] Q. L. Loh and C. Choong , “Three‐Dimensional Scaffolds for Tissue Engineering Applications: Role of Porosity and Pore Size,” Tissue Engineering Part B: Reviews 19 (2013): 485–502. 23672709 10.1089/ten.teb.2012.0437 PMC3826579

[54] J. Zeltinger , J. K. Sherwood , D. A. Graham , R. Müeller , and L. G. Griffith , “Effect of Pore Size and Void Fraction on Cellular Adhesion, Proliferation, and Matrix Deposition,” Tissue Engineering 7 (2001): 557–572. 11694190 10.1089/107632701753213183

[55] L. R. Omrani , Z. Moradi , M. Abbasi , M. J. Kharazifard , and S. N. Tabatabaei , “Evaluation of Compressive Strength of Several Pulp Capping Materials,” Journal of Dentistry 22 (2021): 41–47. 33681422 10.30476/DENTJODS.2020.83964.1063 PMC7921770

[56] N. Golafshan , R. Rezahasani , M. Tarkesh Esfahani , M. Kharaziha , and S. N. Khorasani , “Nanohybrid Hydrogels of Laponite: PVA‐Alginate as a Potential Wound Healing Material,” Carbohydrate Polymers 176 (2017): 392–401. 28927623 10.1016/j.carbpol.2017.08.070

[57] K. T. Huang , Y. L. Fang , P. S. Hsieh , et al., “Zwitterionic Nanocomposite Hydrogels as Effective Wound Dressings,” Journal of Materials Chemistry B 4 (2016): 4206–4215. 32264623 10.1039/c6tb00302h

[58] D. Miao , Y. Gao , B. Shi , et al., “Biobased Adhesive Hydrogels for Wound Management and Tissue Repair: From Materials to Advanced Applications,” APL Bioengineering 10 (2026): 011501. 41743456 10.1063/5.0312916 PMC12931969

[59] C. Shen , J. Wang , G. Li , et al., “Boosting Cartilage Repair With Silk Fibroin‐DNA Hydrogel‐Based Cartilage Organoid Precursor,” Bioactive Materials 35 (2024): 429–444. 38390528 10.1016/j.bioactmat.2024.02.016 PMC10881360

[60] C. Xue , L. Chen , N. Wang , et al., “Stimuli‐Responsive Hydrogels for Bone Tissue Engineering,” Biomaterials Translational 5 (2024): 257–273. 39734705 10.12336/biomatertransl.2024.03.004 PMC11681187

[61] C.‐H. Lin , J. R. Srioudom , W. Sun , et al., “The Use of Hydrogel Microspheres as Cell and Drug Delivery Carriers for Bone, Cartilage, and Soft Tissue Regeneration,” Biomaterials Translational 5 (2024): 236–256. 39734701 10.12336/biomatertransl.2024.03.003 PMC11681182

[62] A. K. Gaharwar , S. M. Mihaila , A. Swami , et al., “Bioactive Silicate Nanoplatelets for Osteogenic Differentiation of Human Mesenchymal Stem Cells,” Advanced Materials 25 (2013): 3329–3336. 23670944 10.1002/adma.201300584

[63] J. K. Bar , A. Lis‐Nawara , and P. G. Grelewski , “Dental Pulp Stem Cell‐Derived Secretome and its Regenerative Potential,” International Journal of Molecular Sciences 22 (2021): 12018. 34769446 10.3390/ijms222112018 PMC8584775

[64] I. M. El‐Sherbiny and M. H. Yacoub , “Hydrogel Scaffolds for Tissue Engineering: Progress and Challenges,” Global Cardiology Science & Practice 2013 (2013): 316–342. 24689032 10.5339/gcsp.2013.38 PMC3963751

[65] Z. Xie , W. Jiang , H. Liu , et al., “Antimicrobial Peptide‐ and Dentin Matrix‐Functionalized Hydrogel for Vital Pulp Therapy via Synergistic Bacteriostasis, Immunomodulation, and Dentinogenesis,” Advanced Healthcare Materials 13 (2024): 2303709. 10.1002/adhm.202303709 38431770

[66] K. Zhang , A. Alaohali , N. Sawangboon , et al., “A Comparison of Lithium‐Substituted Phosphate and Borate Bioactive Glasses for Mineralised Tissue Repair,” Dental Materials 35 (2019): 919–927. 30975482 10.1016/j.dental.2019.03.008 PMC6559152

[67] Y. Kong , X. Hu , Y. Zhong , et al., “Magnesium‐Enriched Microenvironment Promotes Odontogenic Differentiation in Human Dental Pulp Stem Cells by Activating ERK/BMP2/SMADS Signaling,” Stem Cell Research & Therapy 10 (2019): 378. 31823825 10.1186/s13287-019-1493-5 PMC6902488

[68] Y. Zhong , C. Liu , X. Yan , et al., “Odontogenic and Anti‐Inflammatory Effects of Magnesium‐Doped Bioactive Glass in Vital Pulp Therapy,” Biomedical Materials 19 (2024): 045026. 10.1088/1748-605X/ad4ada 38740053

[69] A. Hoppe , N. S. Güldal , and A. R. Boccaccini , “A Review of the Biological Response to Ionic Dissolution Products From Bioactive Glasses and Glass‐Ceramics,” Biomaterials 32 (2011): 2757–2774. 21292319 10.1016/j.biomaterials.2011.01.004

[70] H. Karkehabadi , E. Khoshbin , R. Najafi , and P. Moosavi , “Effects of Dopamine on the Proliferation and Osteo/Odontogenic Differentiation of Stem Cells of the Apical Papilla,” Molecular Biology Reports 52 (2025): 963. 41021070 10.1007/s11033-025-11026-9

[71] C. Wang , S. Wang , K. Li , et al., “Preparation of Laponite Bioceramics for Potential Bone Tissue Engineering Applications,” PLoS ONE 9 (2014): 99585. 10.1371/journal.pone.0099585 PMC4067276 24955961

[72] H. D. Buzoglu , M. Ozcan , O. Bozdemir , et al., “Evaluation of Oxidative Stress Cycle in Healthy and Inflamed Dental Pulp Tissue: A Laboratory Investigation,” Clinical Oral Investigations 27 (2023): 5913–5923. 37642737 10.1007/s00784-023-05203-y

[73] V. Vengerfeldt , R. Mändar , M. Saag , A. Piir , and T. Kullisaar , “Oxidative Stress in Patients With Endodontic Pathologies,” Journal of Pain Research 10 (2017): 2031–2040. 28894386 10.2147/JPR.S141366 PMC5584906

[74] B. Channer , S. M. Matt , E. A. Nickoloff‐Bybel , et al., “Dopamine, Immunity, and Disease,” Pharmacological Reviews 75 (2023): 62–158. 36757901 10.1124/pharmrev.122.000618 PMC9832385

[75] S. Shirawachi , K. Takeda , T. Naruse , et al., “Oxidative Stress Impairs the Calcification Ability of Human Dental Pulp Cells,” BMC Oral Health 22 (2022): 437. 36192671 10.1186/s12903-022-02467-w PMC9531526

[76] S. H. Zaky , M. Shehabeldin , H. Ray , and C. Sfeir , “The Role of Inflammation Modulation in Dental Pulp Regeneration,” European Cells & Materials [Electronic Resource] 41 (2021): 184–193. 33583014 10.22203/eCM.v041a13

[77] P. R. Cooper , I. J. Chicca , M. J. Holder , and M. R. Milward , “Inflammation and Regeneration in the Dentin‐Pulp Complex: Net Gain or Net Loss?,” Journal of Endodontics 43 (2017): S87–S94. 28844308 10.1016/j.joen.2017.06.011

[78] A. Shapouri‐Moghaddam , S. Mohammadian , H. Vazini , et al., “Macrophage Plasticity, Polarization, and Function in Health and Disease,” Journal of Cellular Physiology 233 (2018): 6425–6440. 10.1002/jcp.26429 PMC13482560 29319160

[79] S. He , Y. Du , H. Tao , and H. Duan , “Advances in Aptamer‐Mediated Targeted Delivery System for Cancer Treatment,” International Journal of Biological Macromolecules 238 (2023): 124173. 36965552 10.1016/j.ijbiomac.2023.124173

[80] G. Li , F. Gao , D. Yang , et al., “ECM‐Mimicking Composite Hydrogel for Accelerated Vascularized Bone Regeneration,” Bioactive Materials 42 (2024): 241–256. 39285909 10.1016/j.bioactmat.2024.08.035 PMC11404060

[81] X. Chen , Z. Xu , Y. Gao , et al., “Framework Nucleic Acid‐Based Selective Cell Catcher for Endogenous Stem Cell Recruitment,” Advanced Materials 36 (2024): 2406118. 10.1002/adma.202406118 39543443

[82] K. Pleiko , M. Haugas , V. Parfejevs , et al., “Targeting Triple‐Negative Breast Cancer Cells with a β1‐Integrin Binding Aptamer,” Molecular Therapy Nucleic Acids 33 (2023): 871–884. 37680989 10.1016/j.omtn.2023.08.015 PMC10481362

[83] M. Kim , J. S. Lee , W. Kim , et al., “Aptamer‐Conjugated Nano‐Liposome for Immunogenic Chemotherapy With Reversal of Immunosuppression,” Journal of Controlled Release 348 (2022): 893–910. 35760233 10.1016/j.jconrel.2022.06.039

[84] Z. Hou , S. Meyer , N. E. Propson , et al., “Characterization and Target Identification of a DNA Aptamer That Labels Pluripotent Stem Cells,” Cell Research 25 (2015): 390–393. 25591927 10.1038/cr.2015.7 PMC4349250

[85] S. C. Gopinath , “Methods Developed for SELEX,” Analytical and Bioanalytical Chemistry 387 (2007): 171–182. 17072603 10.1007/s00216-006-0826-2

[86] M. Suzuki , Y. Taira , C. Kato , K. Shinkai , and Y. Katoh , “Histological Evaluation of Direct Pulp Capping of Rat Pulp With Experimentally Developed Low‐Viscosity Adhesives Containing Reparative Dentin‐Promoting Agents,” Journal of Dentistry 44 (2016): 27–36. 26620099 10.1016/j.jdent.2015.11.005

