# Supplementary material for: Aptamer‐Directed Porous DNA Nanocomposite Hydrogel for Active Pulp Preservation: Immunomodulation, Stem Cell Recruitment and Reparative Dentinogenesis
Source: Adv Sci (Weinh). 2026 May 7;13(41):e75497. doi: 10.1002/advs.75497 (PMC13335494; doi:10.1002/advs.75497)
Supplement: Supplementary file 1 — Supporting File: advs75497‐sup‐0001‐SuppMat.docx. [file ADVS-13-e75497-s001.docx]

**Supporting Information**

**Aptamer-Directed Porous DNA Nanocomposite Hydrogel for Active Pulp Preservation: Immunomodulation, Stem Cell Recruitment and Reparative Dentinogenesis**


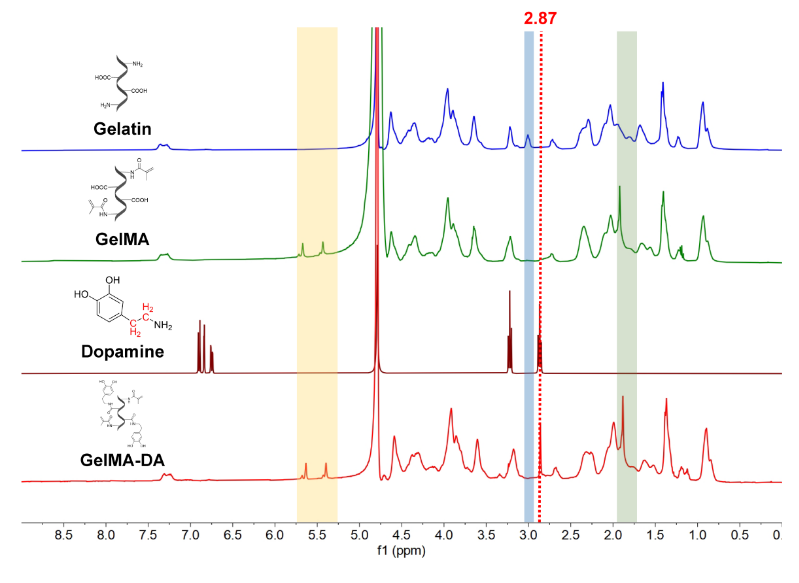


**Figure S1.** ^1^H-NMR spectra of Gelatin, GelMA, Dopamine and GelMA-DA.


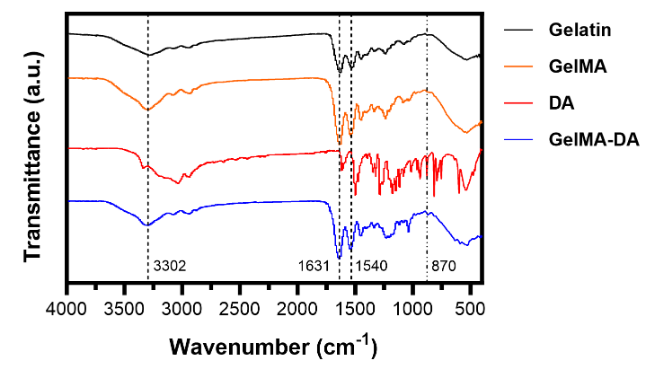


**Figure S2.** FTIR spectra of Gelatin, GelMA and GelMA-DA.


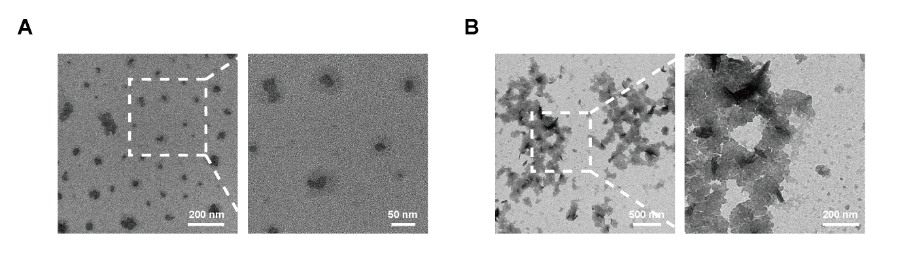


**Figure S3.** TEM images of Laponite clay nanosheets and the DGDL precursor. (A) Representative TEM image of pristine Laponite, displaying its characteristic disc-shaped nanoplatelet structure. (B) Representative TEM image of the DGDL precursor, demonstrating the homogeneous dispersion of Laponite within the polymer matrix without significant aggregation.


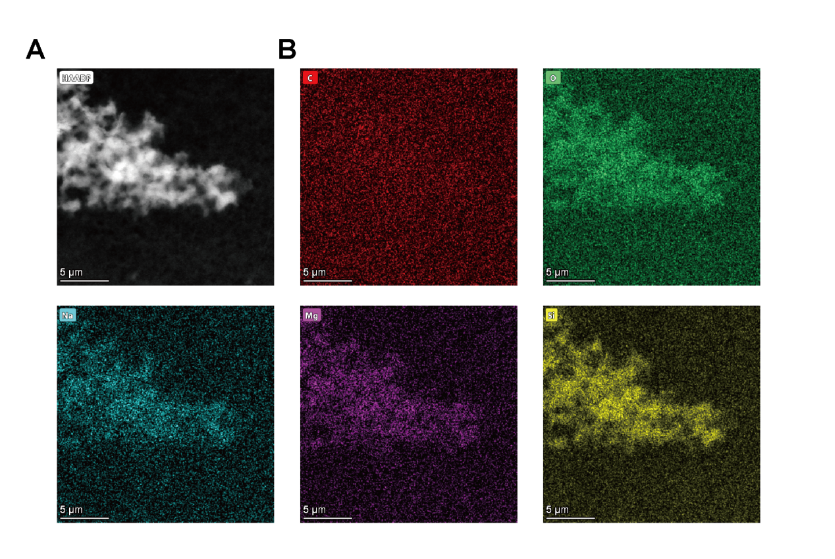


**Figure S4.** TEM-EDS images of the DGDL precursor. The detection of Si and Mg elements, alongside C, O and Na, validates the presence of inorganic Laponite dispersed within the polymer network.


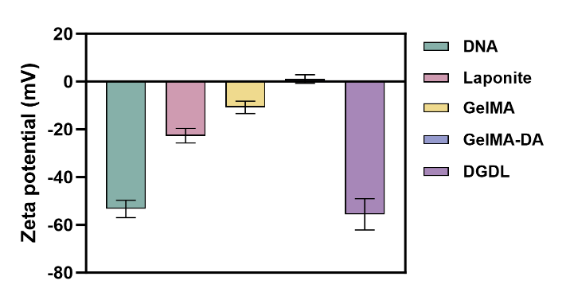


**Figure S5.** Zeta potential values of DNA, Laponite, GelMA, GelMA-DA and the DGDL mixture, indicating the electrostatic characteristics of each component.


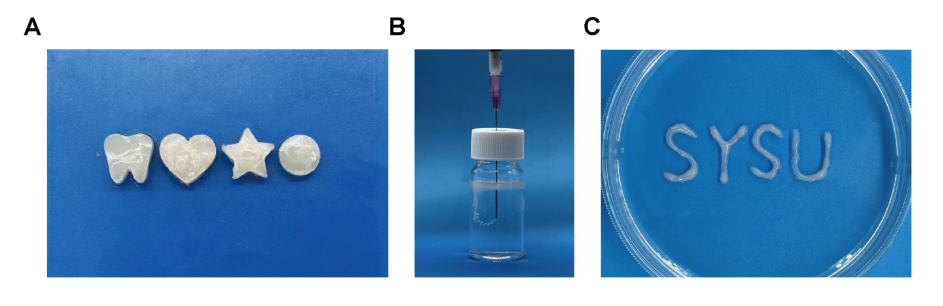


**Figure S6.** Macroscopic evaluation of the shape fidelity and injectability of the DGDL hydrogel.

1. Optical images displaying the hydrogel cured into various geometries (star, heart, circle, and tooth), demonstrating its excellent shape fidelity and ability to form structures with well-defined boundaries. (B, C) Demonstration of the hydrogel's injectability. The hydrogel exhibited smooth extrusion through a standard 24G needle (B) and allowed for the continuous writing of the “SYSU” pattern (C), highlighting its superior flowability and suitability for minimally invasive delivery applications.


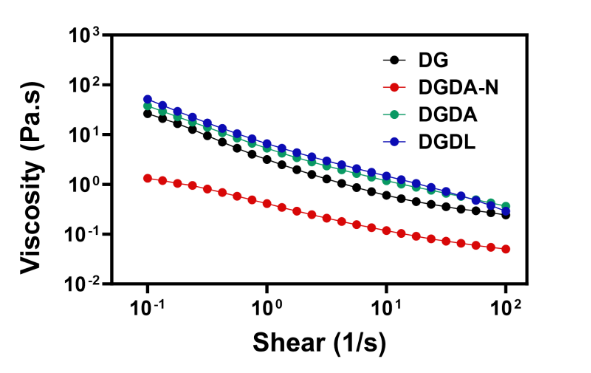


**Figure S7.** Shear-thinning properties of DG, DGDA-N, DGDA and DGDL precursors.


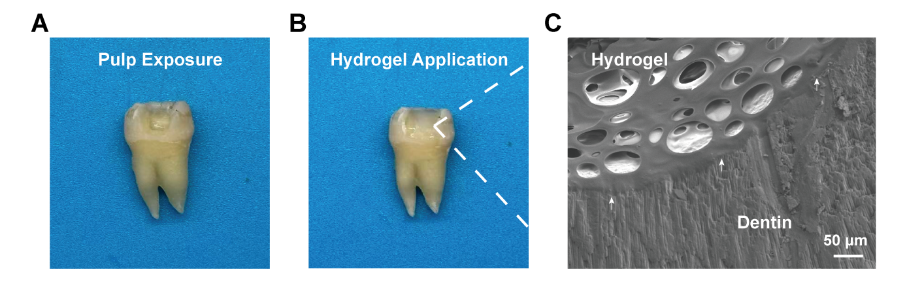


**Figure S8.** Evaluation of the interfacial integration between the DGDL hydrogel and Dentin. (A) A representative model of a human tooth defect with pulp chamber exposure prepared by a fissure bur. (B) Visual observation of the DGDL hydrogel successfully injected and cured within the defected area. (C) SEM image of the cross-sectional interface between the DGDL hydrogel and dentin, showing a seamless integration (white arrows indicate the interface).


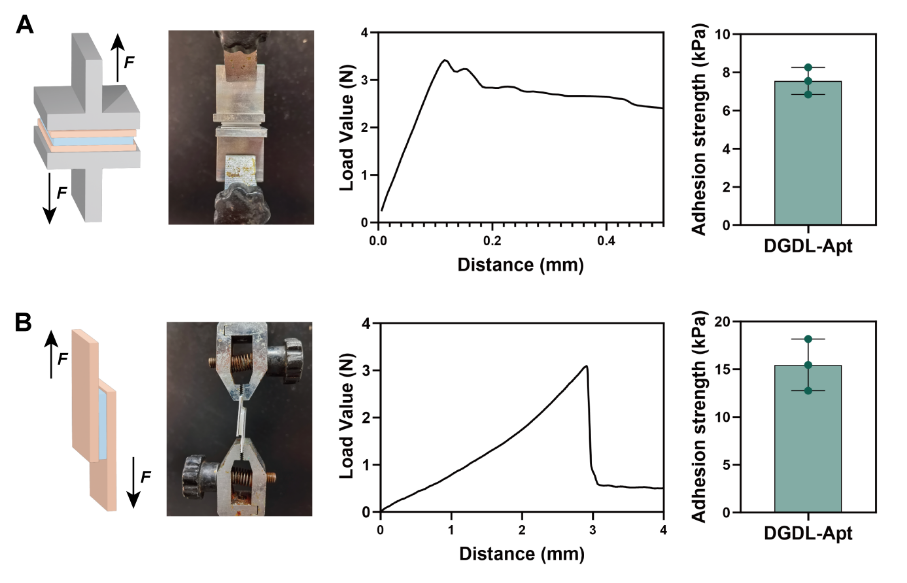


**Figure S9.** Evaluation of the adhesive properties of the DGDL hydrogel on simulated dental substrates. (A) Representative load-distance curve of the tensile adhesion test conducted using ceramic plates. (B) Representative load-distance curve of the lap-shear test performed on ceramic plates.


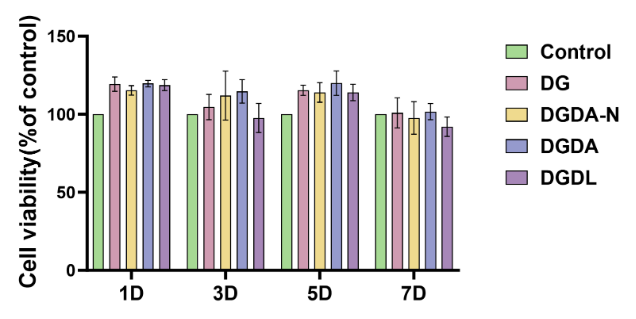


**Figure S10.** Assessment of cell proliferation and cytotoxicity via CCK-8 assay. The viability of dental pulp stem cells (DPSCs) cultured with extracts of DG, DGDA-N, DGDA and DGDL hydrogels was evaluated at days 1, 3, 5 and 7. Data are presented as mean ± SD. (*p* < 0.05)


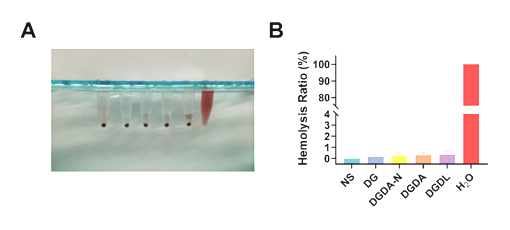


**Figure S11.** Hemolysis test of the hydrogels. (A) Digital photos and (B) hemolysis percentages of the DG, DGDA-N, DGDA and DGDL groups compared to normal saline (NS) and water (H_2_O) controls. (B) The hemolysis percentages of DG, DGDA-N, DGDA, and DGDL hydrogels. Data are presented as mean ± SD.

**Figure S12.** *In vitro* evaluation of the specific cell capture capacity and selectivity of various aptamer-functionalized hydrogels. (A) Representative microscopy images of hDPSCs cultured on non-emulsified hydrogels incorporated with no aptamer (Control), Apt CD29, Apt CD44 or Apt 19S. The observation was performed at 6 hours post-seeding to minimize the interference of non-specific cell adhesion intrinsically driven by the GelMA matrix. Scale bar: 400 μm. (B) Quantitative analysis of the adhered hDPSCs on the corresponding hydrogel surfaces at 6 hours post-seeding. Data represent mean ± SD (n=3), *p< 0.05, ***p < 0.001.


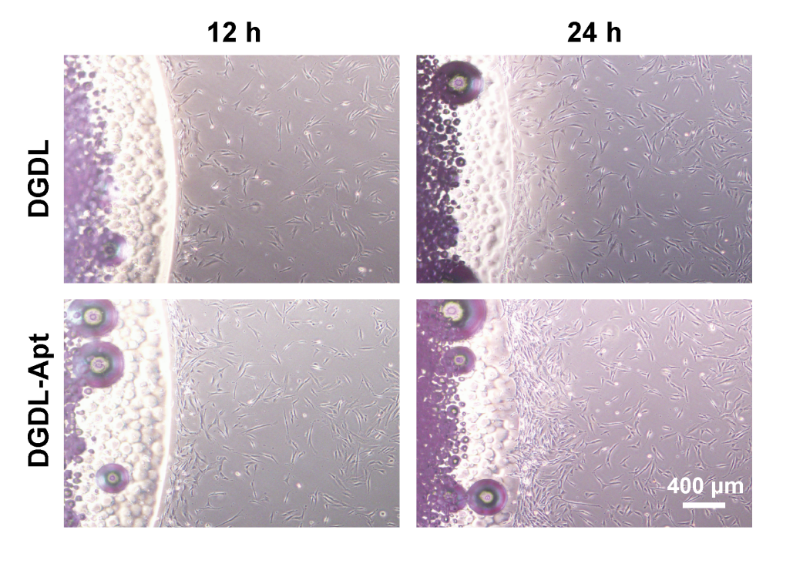


**Figure S13.** *In vitro* targeted recruitment of hDPSCs. Representative microscopy images illustrating the migration of hDPSCs towards the localized DGDL and DGDL-Apt hydrogels at 12 h and 24 h post-seeding. Scale bar: 400 μm.


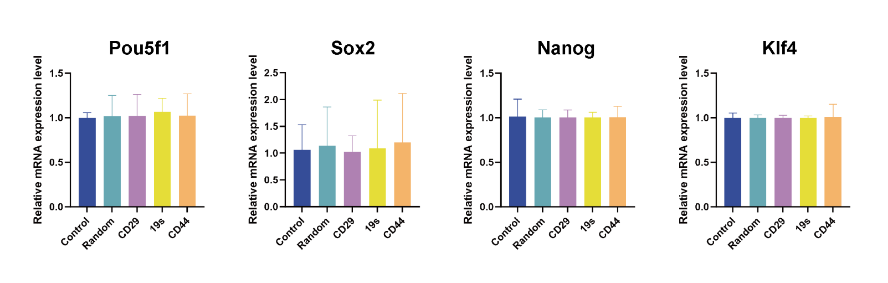


**Figure S14.** qRT-PCR analysis of stemness-related gene expression. Quantitative analysis of Pou5f1, Sox2, Nanog and Klf4 mRNA levels. Data are normalized to GAPDH and presented as fold change relative to the control group.


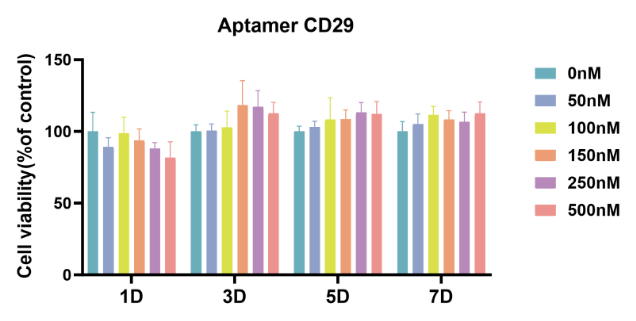


**Figure S15.** Evaluation of cell viability under different CD29 aptamer concentrations. CCK-8 assay results showing the optical density (OD) values of DPSCs cultured with varying concentrations of the aptamer at days 1, 3, 5 and 7. Data are presented as mean ± SD (n = 3).


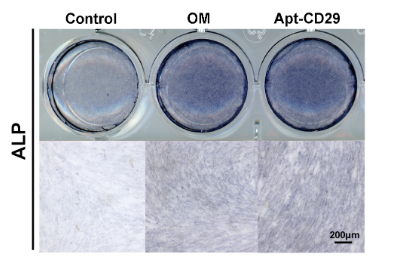


**Figure S16.** Macroscopic and microscopic assessment of odontogenic differentiation. ALP (day 7) and ARS (day 14) staining for Control (basal medium), OM (induction medium), and Apt-CD29 groups.

**Figure S17.** H&E staining images of major organs (heart, liver, spleen, lung, and kidney) harvested 2 months post-surgery.


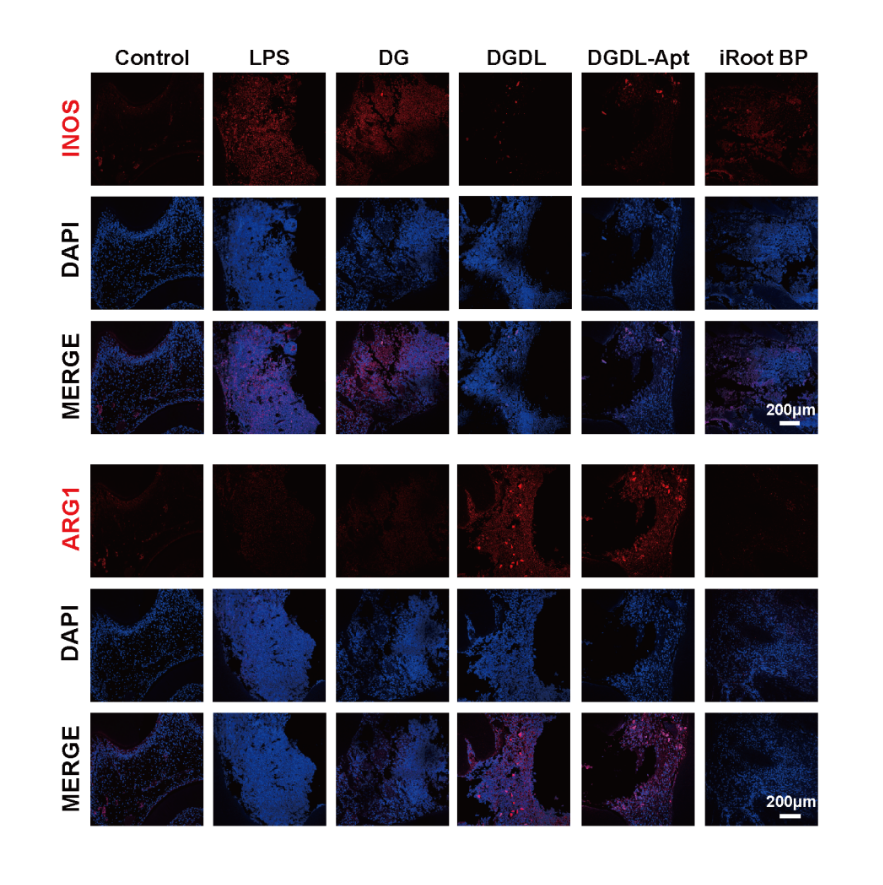


**Figure S18.** *In vivo* assessment of macrophage phenotypes at day 7. Immunofluorescence analysis of iNOS (M1 marker) and Arg-1 (M2 marker) expression in the pulp capping area. Comparison between Control, LPS, DG, DGDL-Apt, and iRoot BP groups indicates distinct macrophage polarization trends. Scale bar represents 200 μm.


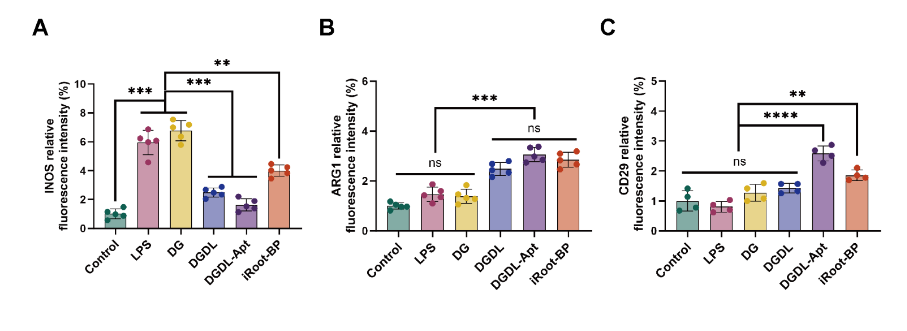


**Figure S19.** Quantitative analysis of immunofluorescence staining intensity at day 3.

(A–C) Quantification of the relative fluorescence intensity per area for (A) iNOS, (B) ARG1, and (C) CD29. Data were normalized to the Control group. Data are presented as mean ± SD (n = 4) (***p* < 0.01, ****p* < 0.001, *****p* < 0.0001)
